# Discovering Genomic Regions Associated with Reproductive Traits and Frame Score in Mexican Simmental and Simbrah Cattle Using Individual SNP and Haplotype Markers

**DOI:** 10.3390/genes14112004

**Published:** 2023-10-27

**Authors:** René Calderón-Chagoya, Vicente Eliezer Vega-Murillo, Adriana García-Ruiz, Ángel Ríos-Utrera, Guillermo Martínez-Velázquez, Moisés Montaño-Bermúdez

**Affiliations:** 1 Faculty of Veterinary Medicine and Zootechnics, National Autonomous University of Mexico, Ciudad de México 04510, Mexico; chagoya.rene@inifap.gob.mx; 2 National Center for Disciplinary Research in Physiology and Animal Improvement, National Institute for Forestry, Agricultural and Livestock Research, Querétaro 76280, Mexico; garcia.adriana@inifap.gob.mx; 3 Faculty of Veterinary Medicine and Zootechnics, Veracruzana University, Veracruz 91710, Mexico; vvega@uv.mx (V.E.V.-M.); ariosu@hotmail.com (Á.R.-U.); 4 Experimental Field Santiago Ixcuintla, National Institute for Forestry, Agricultural and Livestock Research, Nayarit 63570, Mexico; martinez.guillermo@inifap.gob.mx

**Keywords:** genes, genome-wide association study (GWAS), haplotypes, Simbrah, Simmental, single nucleotide polymorphism (SNP)

## Abstract

Reproductive efficiency stands as a critical determinant of profitability within beef production systems. The incorporation of molecular markers can expedite advancements in reproductive performance. While the use of SNPs in association analysis is prevalent, approaches centered on haplotypes can offer a more comprehensive insight. The study used registered Simmental and Simbrah cattle genotyped with the GGP Bovine 150 k panel. Phenotypes included scrotal circumference (SC), heifer fertility (HF), stayability (STAY), and frame score (FS). After quality control, 105,129 autosomal SNPs from 967 animals were used. Haplotype blocks were defined based on linkage disequilibrium. Comparison between haplotypes and SNPs for reproductive traits and FS was conducted using Bayesian and frequentist models. 23, 13, 7, and 2 SNPs exhibited associations with FS, SC, HF, and STAY, respectively. In addition, seven, eight, seven, and one haplotypes displayed associations with FS, SC, HF, and STAY, respectively. Within these delineated genomic segments, potential candidate genes were associated.

## 1. Introduction

Reproductive efficiency is essential for the profitability of beef production systems. High reproductive rates significantly increases the biological and economic efficiency of cow–calf production systems [1]. The main factors that significantly impact reproductive efficiency in cattle include the timing of puberty and first conception, the duration of post-partum anoestrus, and the overall lifetime productivity. This metric encapsulates the combined effects of cow survival and reproductive performance as well as the survival and growth rate of their offspring [2].

Genetic evaluations can expedite improvements in reproductive performance and other traits with low heritability [3]. Additionally, the utilization of molecular markers can enhance the accuracy of heritability estimations. Genotypes obtained via SNP panels have applications in the study of complex traits, the exploration of linkage disequilibrium patterns, QTL mapping, genomic selection, and genetic gain calculation [4]. The availability of individuals genotyped with high-density SNP panels simplifies the identification of genomic regions linked to economically significant traits, improving the detection of QTLs and genetic variation.

While SNPs are commonly used in association analyses, haplotype-based methods offer more comprehensive information. In contrast to SNPs, which can have a maximum of two alleles, haplotype blocks can encompass more than two haplotypes. Haplotype blocks consist of two or more closely located loci, making them prone to co-inheritance due to their high linkage disequilibrium. Haplotype-based methodologies, aimed at enhancing the efficacy of identifying causal haplotypes, are rational, considering that genes operate as coherent gene sets, rather than as individual SNPs within the set. These haplotype-based techniques are anticipated to provide improved control over false positives when compared to single-SNP methods as they concentrate on the complete haplotype block rather than on the individual SNPs within that block [5]. Haplotype construction compresses multiple SNPs into a haplotype locus and optimizes the design of genomic selection and GWAS [6].

The ability to replicate the regions is associated with the economically significant traits reported in other studies [7,8,9,10,11], which enables us to validate these associations as there is a possibility of false positives in the association analyses [12]. Hence, without this validation, the number and identity of the regions and genes influencing the expression of reproductive traits remain uncertain. In beef cattle, this situation has also been emphasized where it is necessary to test these genomic regions to determine whether their use in genomic selection will be beneficial in crossbreeding programs [13].

Also, a multi-breed reference population has the potential to significantly enhance the precision of genomic predictions by enabling breeds with limited reference population data to leverage information from other breeds [14]. Notably, improvements in the accuracy of genomic predictions are achieved when the breeds included in the multi-breed reference population share close genetic relationships [14].

The objective of this study was to compare the results of an association analysis with haplotypes and SNPs for reproductive traits and frame score (FS) in Simmental and Simbrah cattle using the EMMAX, BayesB, and BayesC methods.

## 2. Materials and Methods

### 2.1. Phenotypic Data

The phenotypes employed in the current study were:

- Frame score (FS): Hip height converted to frame score represents a linear measurement employed by cattle producers to assess the potential lean-to-fat ratio of an individual animal within a performance-oriented program. Calculated using the reference tables provided by the Beef Improvement Federation [15].
- Scrotal circumference (SC): For yearling bulls, scrotal circumference measurement entails encircling the broadest section of the scrotum with a scrotal tape while the testicles are in a fully extended state.
- Heifer fertility (HF): Represents the probability that the daughters of a sire have their first calving at the age of 3 years or earlier.
- Stayability (STAY): Indicates the probability that the daughters of a sire, having had a calf before 3 years of age, will have at least a second calf before 6 years of age.

Estimated breeding values (EBV) for all traits were computed using data from the Mexican Simmental-Simbrah Breeders Association, which was subsequently submitted to Mexico’s National Institute for Forestry, Agriculture, and Livestock Research (INIFAP) for the National Genetic Evaluation.

In line with the protocols detailed by Garrick et al. [16], these EBV were deregressed (DEBV). Following the deregression process, males with low DEBV were excluded from the analysis. This resulted in a total of 547 Simmental and 583 Simbrah animals that were considered for further analyses.

### 2.2. Genotypic Data

Blood samples were obtained from each of the animals participating in the study. These samples were individually labeled and subsequently dispatched to Neogen’s GeneSeek Laboratory, located in Lincoln, NE, USA, for the purpose of DNA extraction and genotyping. The GGP Bovine 150 k chip, consisting of 138,962 SNP markers, was employed for genotyping. All SNPs with a call rate lower than 0.95 and a minor allele frequency below 0.05 were excluded. Furthermore, individuals with a call rate lower than 0.95 were removed from the dataset. Following the quality control assessment, a total of 105,129 autosomal SNPs from 967 animals (473 Simmental and 494 Simbrah) were employed for genotype association tests [17].

Haplotype blocks were defined as regions smaller than 200 kb, with at least 90% of the comparisons between SNPs within the block in “strong LD”. The 90% confidence interval for LD was established between 0.70 and 0.98. A total of 16,651 haplotype blocks (each block had between 2 and 48 SNPs) containing 57,202 haplotypes were inferred and used for the association analysis. Quality control and haplotypes were determined with Plink 1.07 [18].

### 2.3. Association Analyses

A comparison was made between haplotypes and SNPs for reproductive traits and FS in Simmental and Simbrah cattle. The association analyses were carried out separately for each breed, combining the information of both breeds (joint analysis). Two different approaches were used, a single-marker GWAS and two Bayesian GWAS.

#### 2.3.1. Single-Marker GWAS

For the single-marker GWAS, EMMAX was implemented using SNP & Variation Suite 7 (Golden Helix, Inc., Bozeman, MT, USA); this is a single-locus mixed model GWAS approach that fits a genomic relationship matrix to account for genetic covariance among animals [19]. In single-locus GWAS, one primary concern is the elevated false positive rate. To mitigate this concern, the Bonferroni correction is commonly employed in single-locus models. We set the false discovery rate to 0.05 to correct for multiple testing. The response variables used were the corrected phenotypes (y-Xb). In the case of this data type, the Mixed Model approach is highly suitable. It allows us to account for relatedness by incorporating the random effects component of the model, utilizing a kinship matrix (IBS), and also enables the inclusion of supplementary fixed effects (such as Breed for joint analyses).

The *p*-values for the Bonferroni-corrected thresholds at the suggestive and 5% genome-wide significance levels were determined by dividing 1 and 0.05, respectively, by the total number of markers utilized in the GWAS. The concept of the suggestive level was originally introduced by Lander and Kruglyak [20] and signifies the threshold at which, assuming the null hypothesis, one false positive is anticipated per genome scan [21]. Following the analysis, none of the SNP or haplotype markers surpassed the significance thresholds at the genome-wide level in both the Simmental and Simbrah data analyses as well as in the joint-data analysis. Consequently, the suggestive threshold level was employed as a reference to identify associations between the markers and economically significant traits.

#### 2.3.2. Bayesian GWAS

The Bayesian models were implemented via the BGLR statistical package of the R program [22]. Two a priori finite mixture models were employed: one comprising a mass point at zero and a Gaussian density, referred to as BayesC [23], and another featuring a mass point at zero along with a scaled t density, known as BayesB [24]. By assigning a nonzero prior probability for the marker effect to equal zero, the principles underlying BayesB and BayesC have the capacity to initiate variable selection.

For a continuous response ($y_{i};\;i=1,\ldots ,\;n$), the data equation is formulated as follows: $y_{i}=\eta_{i}+\varepsilon_{i}\;$, where $\eta_{i}$ represents a linear predictor, the expected value of $y_{i}$ given the predictors, and $\varepsilon_{i}$ denotes the residuals. These residuals are independent and conform to a normal distribution with a mean of zero and a variance $w_{i}^{2}\sigma_{\varepsilon }^{2}$. The linear predictor serves as the conditional expectation function and is structured as:

(1) $$\eta =1\mu +X_{1}\beta_{1},$$

(2) $$\eta =1\mu +X_{1}\beta_{1}+X_{2}\beta_{2},$$

*μ* represents the intercept and $X_{j}$ stands for the design matrices for the predictors, denoted as $X_{j}=\left\{x_{ijk}\right\}$, where $x_{ijk}$ is the marker in the region *j* from the breed *k* of the individual *i* and $\beta_{j}$ are vectors of the effects associated with the columns of $X_{j}$. For the Simmental and Simbrah analysis model (1) $X_{1}$ is the matrix with marker genotypes, and $\beta_{1}$ is the corresponding vector of marker effects. For the joint analysis model (2), $X_{1}$ is a design matrix for the effects of breed, $\beta_{1}$ is the corresponding vector of effects, $X_{2}$ is the matrix with marker genotypes, and $\beta_{2}$ is the corresponding vector of marker effects. Bringing together these assumptions, we obtain the following conditional distribution of the data:

$$p\left(y|\theta \right)=\prod_{i=1}^{n}N\left(y_{i}|\mu +\sum_{j=1}^{J}\sum_{k=1}^{K_{j}}x_{ijk}\beta_{jk},\sigma_{\varepsilon }^{2}w_{i}^{2}\right)$$

where θ represents the collection of unknowns, including the intercept, regression coefficients $\left(\beta_{jk}\right),$ and the residual variance. The analysis employs Markov chain Monte Carlo (MCMC) methods to calculate posterior mean estimates of marker effects and variances. The MCMC chains consist of 100,000 iterations, with the initial 25,000 samples designated for burn-in.

The Gelman–Rubin diagnostic was employed to evaluate the Bayesian models, which evaluates MCMC convergence by analyzing the difference between multiple Markov chains. The convergence is assessed by comparing the estimated between-chains and within-chain variances for each model parameter. To facilitate this process, adjustments were made to the potential scale reduction factor, with the estimated degrees of freedom (d) serving as the pivotal parameter for this adaptation. This adjustment relies on a Student-t approximation for posterior inference derived from the simulations [22],

$${\hat{R}}_{c}=\frac{d+3}{d+1}\hat{R}$$

Significant disparities in these variances signal non-convergence (${\hat{R}}_{c}>1.1)$. In cases where the model failed to converge, the number of iterations and burn-ins were doubled. After estimating marker effects, 95% confidence intervals and posterior probabilities for these effects were calculated. Marker effects that equal zero signify the absence of any marker’s influence. To select those markers associated with a trait, posterior probabilities and confidence intervals were used.

### 2.4. Candidate Gene Annotations and Previously Reported QTL

The SNP and haplotypes associated were mapped at 100 kb intervals on both sides, given the average LD (r^2^ = 0.2) previously observed in beef cattle [13]. Potential candidate genes were identified and linked to the associations using the latest genetic annotations from ENSEMBL (release 104), based on the *Bos taurus* ARS-UCD1.2 genome assembly [25]. A gene was considered a candidate if at least one marker window overlapped with it.

Furthermore, a search was conducted for QTLs previously associated with these SNP and haplotype windows within the same regions. The identified QTLs were compared to those recorded in the Cattle QTL Database [26].

## 3. Results

**Figure 1:**
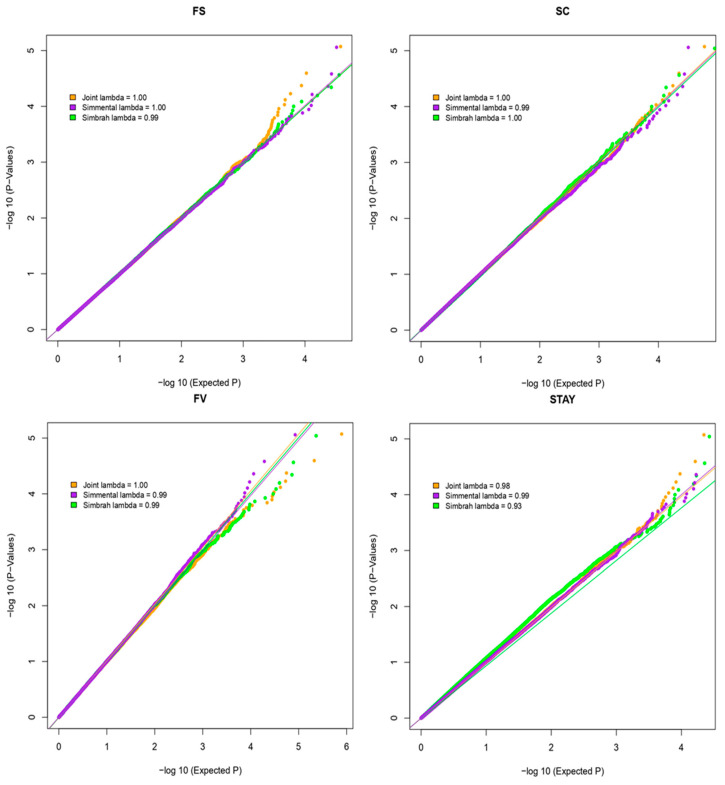
Quantile–Quantile (Q-Q) plot with the haplotype association analysis for frame score (FS), scrotal circumference (SC), heifer fertility (HF), and stayability (STAY) using the EMMAX method in Simmental, Simbrah, and Joint Analysis.

Except for the analysis conducted on STAY in Simbrah cattle, which generated no identified associated markers, the Quantile–Quantile (Q-Q) plots did not reveal any substantial deviations (Figure 1). The close alignment of observed values with the expected values is evident, with most data points residing along or in close proximity to the central line between the x-axis and the y-axis. This absence of early divergence between expected and observed values indicates the absence of population stratification [27].

**Figure 2:**
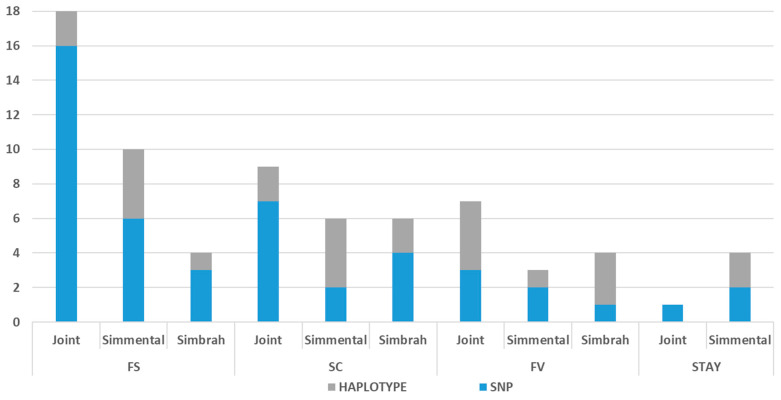
SNP markers and haplotypes associated with frame score (FS), scrotal circumference (SC), heifer fertility (HF), and stayability (STAY) in Simmental, Simbrah, and Joint Analysis.

Figure 2 displays a bar plot representing the distribution of SNP markers and haplotypes associated with FS, SC, HF, and STAY in Simmental, Simbrah, and the Joint Analysis. Also, by using the Cattle QTL Database [26], 118, 74, 45, and 28 QTLs previously described were found within the SNP windows for FS, SC, HF, and STAY, respectively; also, 50, 83, 63, and 16 QTLs previously described were found within the haplotype windows for FS, SC, HF, and STAY, respectively (Table S1). For all the traits, QTLs associated with conformation, health, meat, carcass, milk, and reproduction traits in cattle were found. Over the database ARS-UCD1.2, 18, 11, 6, and 2 genes were found within the SNP windows for FS, SC, HF, and STAY, respectively; also, 2, 22, 7, and 1 QTLs previously described were found within the haplotype windows for FS, SC, HF, and STAY, respectively (Table S1).

### 3.1. Frame Score

In the joint association analysis of FS with SNPs, 15 associated regions were located on chromosomes 4, 5, 6, 7, 8, 11, 16, 17, and 22 (Table S2). Only one marker located on chromosome 17 was associated with all models (BayesB, BayesC, and EMMAX). Three regions were identified with the BayesC model on chromosomes 6, 7, and 8, and the remaining regions were linked with the Bayesian models.

In the Simmental data analysis with SNPs, six associated regions were found on chromosomes 2, 17, 19, and 20 (Table S2). The associated regions on chromosomes 17 and 19 were determined using the BayesC model, and the remaining regions were determined with both the BayesB and BayesC models.

For Simbrah cattle, associated regions were found on chromosomes 3, 17, and 18 (Table S2). There was only one associated region using the BayesB model on chromosome 17. The remaining regions were found with both BayesB and BayesC models.

**Figure 3:**
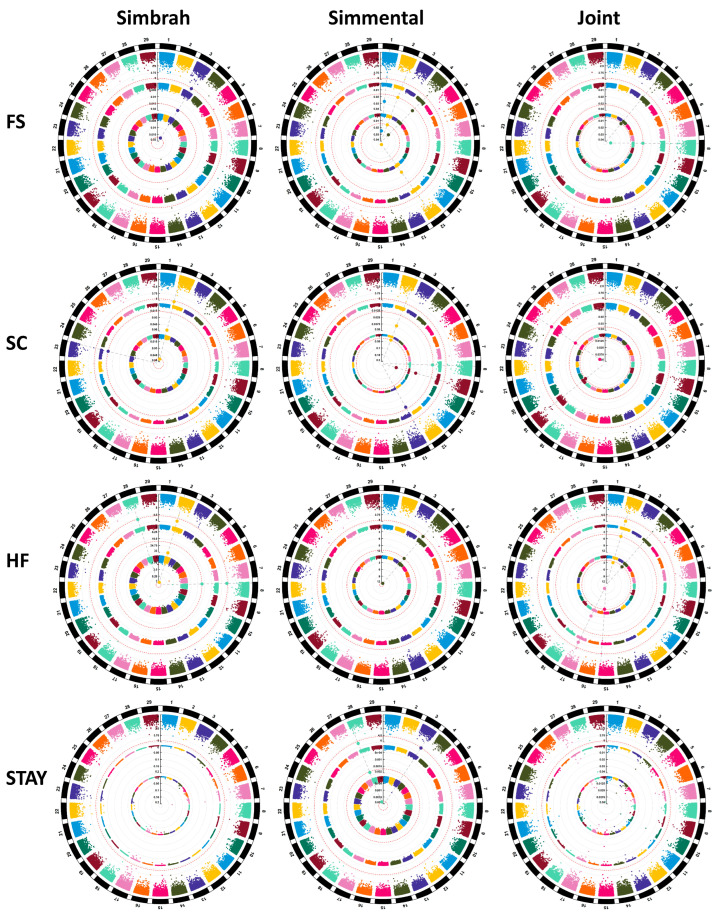
Circle Manhattan Plot with the haplotype association analysis for frame score (FS), scrotal circumference (SC), heifer fertility (HF), and stayability (STAY) with the EMMAX (outer circle, −log_10_ *p*-values), BayesB (middle circle, posterior probabilities), and BayesC (inner circle, posterior probabilities) model in Simbrah, Simmental, and joint cattle. The associated haplotypes are above the red line with big dots in each analysis.

In the haplotype association analysis with the joint analysis, two regions were identified on chromosomes 4 and 8, both with the BayesC model (Table S3). In addition, the region located on chromosome 8 was associated with the BayesB model (Figure 3).

In the haplotype analysis of the Simbrah cattle, only one region on chromosome 3 was identified with the three models (Table S3). In Simmental cattle, four regions were located on chromosomes 1, 2, 4, and 12 with the two Bayesian models (Figure 3).

By contrasting the regions associated with SNPs and haplotypes, we found two regions in common in the joint analysis (Table S3). For the individual analyses of Simmental and Simbrah, one region was found to be common in each breed (Figure 3).

### 3.2. Scrotal Circumference

According to the joint association analysis of scrotal circumference with SNPs, seven associated regions were located on chromosomes 1, 3, 6, 10, and 17 (Table S2). With the three models (BayesB, BayesC, and EMMAX), only one marker was associated with chromosome 10. The other regions were associated with BayesB and BayesC, except the one located on chromosome 1, which was only associated with BayesC.

Two associated regions were found on chromosomes 10 and 13 in the association analysis with SNPs for Simmental cattle (Table S2). All models determined the associated region on chromosome 10, and Bayesian models determined the remaining regions.

There were associated regions on chromosomes 6, 10, 17, and 23 for Simbrah cattle (Table S2). On chromosomes 17 and 23, there were two regions associated with BayesC. The remaining regions were detected by BayesB and BayesC.

On chromosomes 19 and 25, the joint analysis revealed two haplotype associated regions (Table S3). Each of the three models determined the associated region on chromosome 25, and the BayesC model determined the remaining region (Figure 3).

The haplotype analysis of Simbrah cattle identified two regions on chromosomes 2 and 23 (Table S3). With all models, we determined the associated region on chromosome 2, and BayesB determined the remaining region (Figure 3).

Four regions were found on chromosomes 2, 8, 9, and 13 of Simmental cattle (Table S3). Chromosomes 8 and 13 were associated with the BayesB model. The remaining regions were found with both BayesB and BayesC models (Figure 3).

By contrasting the regions associated with SNPs and haplotypes, we found no regions in common in the joint analysis. For the individual analyses of Simmental and Simbrah, one region was found to be common in each case. In addition, there was a region in the SNP analysis in common between Simmental and Simbrah with the joint analysis in chromosomes 10 and 6, respectively.

### 3.3. Heifer Fertility

The joint association analysis of heifer fertility with SNPs identified four associated regions on chromosomes 4, 17, and 18 (Table S2). With the three models (BayesB, BayesC, and EMMAX), only one region on chromosome 17 was associated. On chromosome 4, BayesC identified two regions, while BayesB and BayesC were linked to the remaining regions.

In the SNP association analysis for Simmental cattle, two associated regions were found on chromosomes 4 and 21 (Table S2). The associated region on chromosome 4 was determined using the BayesC model, and the remaining region was determined by all models. As for Simbrah cattle, an associated region was found on chromosome 17 with all models.

Based on the haplotype association analysis with the joint analysis, four regions were identified on chromosomes 2, 4, 15, and 17 (Table S3). Two regions were identified with the BayesC model on chromosomes 4 and 15, and the remaining regions were linked with all models (Figure 3).

The haplotype analysis of Simbrah cattle identified only three regions on chromosomes 2, 8, and 28 (Table S3). All regions were associated with the EMMAX model. BayesB was linked with chromosomes 2 and 8. The BayesC model also determined the region associated with chromosome 2. Simmental cattle showed a region on chromosome 4 with all models (Figure 3).

Comparing the regions associated with SNPs and haplotypes, we found one region in common. Additionally, the Simbrah and joint analyses shared a region on chromosome 17.

### 3.4. Stayability

In the joint association analysis of STAY with SNPs, one associated region was located on chromosome 21 with the BayesC model (Table S2). In Simmental cattle, associated SNPs were found on chromosomes 1 and 28 using the two Bayesian models (Table S2). In addition, the EMMAX model was linked to the chromosome 28 region. The EMMAX model identified an additional region on chromosomes 3 in the Simmental cattle haplotype analysis. In Bayesian models, the region associated with chromosome 28 was located as well.

Our analysis of Simmental individual analyses of chromosome 28 revealed a region common to both haplotypes and SNPs (Table S2).

## 4. Discussion

### 4.1. Frame Score

In the Simmental data analysis, within the SNP windows on chromosome 2, QTLs were previously associated with a body form composite index in Holstein cattle; this index contemplates the height of the animal, which is a measure that is also used to calculate FS [28]. Also, inside of an SNP window on chromosome 19, three different traits were previously associated, these included a QTL for body weight (yearling) in Angus cattle [8], which is a trait correlated with FS [29]. In the same way, in the same study, they associated this region with another QTL for SC [8], which is also related to FS [29], and stature through an association analysis in Holstein cattle [30]. Finally, within a haplotype window, a QTL was also associated with body weight (yearling) in Angus cattle [8].

From the results of the joint model, several coincidences were found within the regions associated with FS, with the databases of QTLs. On chromosome 5 and 7, associations with SC in Angus cattle [8] were found, which is a trait correlated with FS [29]. Also, QTLs associated with body weight (yearling) were found within chromosome 6 and 22; the first one was in crossbred beef cattle [31] and the second in Angus cattle [8], and as already mentioned, this is a trait correlated with FS [29].

The ligand-dependent nuclear receptor corepressor-like (LCORL) gene on chromosome 6 has been reported to be associated with body frame size (height) at puberty in Japanese Black and Charolais x German Holstein cattle [32]; it is the same region associated in the present work with FS (Table S1).

### 4.2. Scrotal Circumference

In the Simbrah data analyses, two QTL associated with body weight (yearling) inside of the haplotypes windows were found on chromosome 2 in Angus × Brahman cattle [33] and 23 in Angus cattle [8] In the same region of chromosome 23, a QTL associated with SC in Angus cattle was found [8].

In Simmental cattle, associations with body weight (yearling) were found within two haplotype windows on chromosomes 8 in crossbreed cattle [31] and 13 in Angus × Brahman cattle [34]. The same region of chromosome 13 was found with the SNP analysis. In addition, within an SNP window, two regions associated with SC and sexual precocity [7] in tropically adapted breeds were found.

In the joint-data analysis, associations were found in two SNPs windows on chromosome 1 in Angus × Brahman cattle [34] and 17 in Angus cattle [8] associated with body weight (yearling). On chromosome 10, associations with SC and sexual precocity were reported; this is the same region found in the current Simmental data analysis. For haplotypes windows, on chromosome 19, two QTLs associated with SC in Angus cattle [8] and the ovulation rate [35] were found. Finally, a haplotype window on chromosome 25 was found in the same region as a QTL associated with body weight (yearling) in Angus cattle [8].

In Iranian Holstein cattle [36], on chromosome 13, the recombination signal binding protein for the immunoglobulin kappa J region-like (RBPJL) gene associated with calving to first service was identified, which is the same gene that was associated in the present work with SC (Table S1).

### 4.3. Heifer Fertility

In the Simmental data analysis, within the SNP windows on chromosome 21, QTLs were previously reported to SC in Angus cattle [8], which is a trait correlated with FS [29]. From the results of the joint model, in the SNP window on chromosome 18, a QTL was associated with the pregnancy rate and length of productive life in Holstein cattle [37]; also, on chromosome 15 within a haplotype window, a QTL associated with age at puberty in crossbreed cattle was reported [38].

In Iranian Holstein cattle [36], on chromosome 2, the ADP ribosylation factor-like GTPase 5A (ARL5A), calcium voltage-gated channel auxiliary subunit *β* 4 (CACNB4) gene associated with calving to first service was identified, which is the same gene that was associated in the present work with HF (Table S1).

RUN Domain Containing 3B (RUNDC3B) on chromosome 4 had been reported to the retained placenta signals with associations related to milk production, productive life, and health and reproduction traits, including calving ease and stillbirth [30,39]. In Holstein cattle on chromosome 4, the Inner Mitochondrial Membrane Peptidase 2-Like Gene (IMMP2L) had a negative effect on the cow conception rate [40]; in mouse, homozygous IMMP2L females were infertile due to defects in folliculogenesis and ovulation, whereas mutant males were severely subfertile due to erectile dysfunction [41].

In goats, the expression of the CKLF-like MARVEL transmembrane domain-containing 2 (CMTM2) gene on chromosome 18 was more elevated in the ovaries of multiple prolific goats at first kidding compared to non-prolific goats [42].

### 4.4. Stayability

In the Simmental data analysis, within the SNP windows on chromosome 1, QTLs were previously reported for the interval to first estrus after calving in Holstein cattle [43]. With the severe body condition loss, there can be longer intervals to the first ovulation and first estrus, lower first service conception rates, and more days open [44]. On chromosome 28 in the same region that an SNP and haplotype window were found, a QTL was previously associated to the pregnancy rate [45].

In Costeño con Cuernos cattle from Colombia, on chromosome 21, the cathepsin H (CTSH) gene associated with age at first calving was identified [46], which is the same gene that was associated in the present study with STAY (Table S1).

### 4.5. Single-Marker GWAS and Two Bayesian GWAS

As shown in the results, none of the SNPs crossed the threshold level at the genome level for the EMMAX model. Nonetheless, this is not an isolated case, as in many studies, single locus GWAS models, such as EMMAX, did not significantly exceed the threshold of significance, so they resort to other methodologies [47]. Although the whole concept of significant thresholds should not be misinterpreted to assume causality and reproducibility of SNP effects [48], it is a useful concept to preselect and differentially weigh SNPs in a genomic prediction between breeds [49].

According to our dataset, while using a single marker with EMMAX makes it more stringent to assert significance, employing Bayesian models offers a superior understanding of how LD affects the outcome. However, as Legarra et al. [50] mentions, the strength of the evidence that combines information from several consecutive markers increases with Bayesian models and decreases with EMMAX. These approaches could benefit frequentist models by offering an improved mixture of evidence among markers. Yet, their suitability for primary detection remains uncertain due to the absence of clearly defined rejection thresholds.

In GWAS, a frequent issue arises when the number of parameters (*p*) is significantly larger than the number of samples (*N*). Consequently, conventional methods of maximizing the likelihood based on effect sizes often result in severe overfitting. To address this problem, a common approach is to introduce a regularization term into the log likelihood, encouraging many components of *β* (effect sizes) to approach zero. From a Bayesian perspective, this can be viewed as maximizing the posterior distribution *p*(*β*|X) [51]. This advantage of Bayesian models over single locus models allows for better handling of overfitting in GWAS.

### 4.6. SNP- and Haplotype-Based Association Analyses

In the present study, we identified several significant SNPs and favorable haplotypes using SNP and haplotype-GWAS approaches, respectively, that regulate FS, SC, HF, and STAY in Simmental and Simbrah cattle. The idea of utilizing haplotype-based GWAS has been suggested as an additional method to enhance the advantages gained from LD [52]. This is because these studies have the potential to offer a new perspective on genetic factors that are not identified via the analysis with SNPs, which allows us to investigate the genetic architecture of traits of economic interest [52].

SNP- and Hap-window approaches to identify important genomic regions accounted for LD and better characterized QTL than the individual effects [53]. For all traits, markers were found in regions previously associated with genes and QTLs for production, reproduction, health, and conformation traits. However, it is of greater interest to focus on the QTLs that are correlated with each trait.

Association analyses have identified many common genetic variants associated with traits of interest, but these variants typically have small effect sizes and account for only a small fraction of the heritability of most traits. This missing heritability revealed in many well-powered analyses associations suggests that there are many important architectural details of traits that remain to be discovered. However, an association that explains only a small proportion of heritability may nonetheless provide valuable biological insights [54]; therefore, the lack of overlapping genomic regions identified for shared traits between Simmental and Simbrah cattle suggests that these breeds have distinct genetic architectures for these traits.

Our study provides new insights into the genetic basis of reproductive traits and FS in Simmental and Simbrah cattle. We identified positional candidate genes with functional evidence that could improve bull genomic prediction for these traits. It has been proven that GWAS-selected sequence variants improve genomic prediction reliability [14], which could have a major impact on the beef cattle industry as it would allow producers to breed more productive and efficient animals.

## 5. Conclusions

A total of 23, 13, 7, and 2 SNPs were associated with FS, SC, HF, and STAY, respectively. Also, seven, eight, seven, and one haplotypes were associated with FS, SC, HF, and STAY, respectively. Through these regions, we identified possible candidate genes for the different traits used in this study. These regions may be useful in Mexican Simmental and Simbrah cattle. No regions were found to be common between Simmental and Simbrah cattle, which indicate that we are dealing with two completely different genetic architectures.

## Data Availability

The data presented in this study are available upon request from the corresponding author.

## Supplementary Materials

The following supporting information can be downloaded at: https://www.mdpi.com/article/10.3390/genes14112004/s1, Table S1: Traits and candidate genes previously identified within the regions associated with reproductive traits and frame score in Simmental and Simbrah cattle; Table S2: SNPs associated with reproductive traits and frame score with the models BAYESB, BAYESC and EMMAX in Simmental and Simbrah cattle; Table S3: Haplotypes associated with reproductive traits and frame score with the models BAYESB, BAYESC, and EMMAX in Simmental and Simbrah cattle.
