# Supplementary material for: Discovering Genomic Regions Associated with Reproductive Traits and Frame Score in Mexican Simmental and Simbrah Cattle Using Individual SNP and Haplotype Markers
Source: Genes (Basel). 2023 Oct 27;14(11):2004. doi: 10.3390/genes14112004 (PMC10671695; doi:10.3390/genes14112004)
Supplement: Supplementary file 1 [file genes-14-02004-s001.zip › genes-2627363-supplementary.pdf]

Supplementary

Table S1. Traits and genes previously described within the regions associated with reproductive traits and frame score in Simmental and Simbrah cattle.

Trait	Chr [a]	Marker	Position	Traits previously associated	Genes
SC	1	BovineHD0100007238	24964189	Exterior= Rump angle (PUBMED_ID= 12605852), Udder cleft (PUBMED_ID= 16167984), Teat length (PUBMED_ID= 15377635), Meat_and_Carcass= Marbling score (PUBMED_ID= 20477797), Oleic acid content (PUBMED_ID= 20477785), Milk= Milk protein yield (PUBMED_ID= 9691050), Milk yield (PUBMED_ID= 7713441), Milk protein yield (PUBMED_ID= 7713441), Milk fat percentage (PUBMED_ID= 14762092), Milk fat yield (PUBMED_ID= 11178740), Production= Height (mature) (PUBMED_ID= 20477797), Body weight (birth) (PUBMED_ID= 20477797), Reproduction= Calving ease (PUBMED_ID= 20477797), Non-return rate (PUBMED_ID= 15377635),	ROBO2
SC	1	BovineHD0100020122	69664149	Health= Infectious bovine keratoconjunctivitis susceptibility (PUBMED_ID= 17093209), Meat_and_Carcass= Oleic acid content (PUBMED_ID= 20477785), Linoleic acid content (PUBMED_ID= 20416790), Linolenic acid content (PUBMED_ID= 20416790), Polyunsaturated fatty acid content (PUBMED_ID= 20416790), Marbling score (PUBMED_ID= 20477797), Carcass weight (PUBMED_ID= 12926775), Fat thickness at the 12th rib (PUBMED_ID= 17894565), Milk= Milk alpha-casein percentage (PUBMED_ID= 19397519), Production= Body weight (yearling) (PUBMED_ID= 12926775), Body weight (slaughter) (PUBMED_ID= 12926775), Body weight (weaning) (PUBMED_ID= 20477797), Reproduction= Fertility treatments (PUBMED_ID= 19841231), Conception rate (PUBMED_ID= 12605852),	SLC12A8, ZNF148
SC	2	HAPLOTYPE	41538841-41567719	Exterior=Udder depth (PUBMED_ID= 17433017), Teat placement (PUBMED_ID= 12605852), Dairy form (PUBMED_ID= 16167984), Health=Immunoglobulin G level (PUBMED_ID= 21138580), Somatic cell score (PUBMED_ID= 25288516), Meat_and_Carcass=Lung percentage (PUBMED_ID= 20477785), Fat thickness at the 12th rib (PUBMED_ID= 14677852), Yield grade (PUBMED_ID= 14677852), Milk=Milk fat yield (PUBMED_ID= 17433017), Milk yield (PUBMED_ID= 12778594), Milk protein yield (PUBMED_ID= 16167984), Milk fat percentage (PUBMED_ID= 14762090), Milk fat yield (PUBMED_ID= 9691050), Milk kappa-casein percentage (PUBMED_ID= 27485317), Production=Body weight (birth), (PUBMED_ID= 14677852), Chest depth (PUBMED_ID= 12605852), Body weight (mature), (PUBMED_ID= 20477797), Body weight (birth), (PUBMED_ID= 20477797),	
SC	2	HAPLOTYPE	5747611-5759652	Exterior=Udder depth (PUBMED_ID= 17433017), Udder attachment (PUBMED_ID= 16230715), Strength (PUBMED_ID= 16230715), Meat_and_Carcass=Longissimus muscle area (PUBMED_ID= 17596127), Retail product yield (PUBMED_ID= 9498354), Longissimus muscle area (PUBMED_ID= 9498354), Yield grade (PUBMED_ID= 9498354), Marbling score (PUBMED_ID= 9498354), Fat thickness at the 12th rib (PUBMED_ID= 9498354), Kidney, pelvic, and heart fat percentage (PUBMED_ID= 9498354), Conjugated linoleic acid content (PUBMED_ID= 17894565), Beef flavor intensity (PUBMED_ID= 17894565), Monounsaturated fatty acid content (PUBMED_ID= 17894565), Oleic acid to stearic acid ratio (PUBMED_ID= 17894565), Carcass weight (PUBMED_ID= 22303340), Lean meat yield (PUBMED_ID= 30290764), Yield grade (PUBMED_ID= 30290764), Longissimus muscle area (PUBMED_ID= 31931697), Milk=Milk fat yield (PUBMED_ID= 16167984), Milk sodium content (PUBMED_ID= 33824377), Production=Body weight (weaning), (PUBMED_ID= 22303340), Body weight (birth), (PUBMED_ID= 9498354), Thurl width (PUBMED_ID= 16230715), Body weight (yearling), (PUBMED_ID= 22303340), Body weight (slaughter), (PUBMED_ID= 22303340), Calving ease (PUBMED_ID= 26065883),	NEMP2
SC	3	BovineHD0300004685	14395023	Health= Somatic cell count (PUBMED_ID= 11845286), Meat_and_Carcass= Marbling score (PUBMED_ID= 14677852), Longissimus muscle area (PUBMED_ID= 20477797), Carcass weight (PUBMED_ID= 20477797), Shear force (PUBMED_ID= 33101375), Milk= Milk protein percentage (PUBMED_ID= 12605852), Milk protein percentage (PUBMED_ID= 27287773), Production= Body weight (weaning) (PUBMED_ID= 20477797), Body weight (mature) (PUBMED_ID= 20477797), Body weight (birth) (PUBMED_ID= 20477797), Reproduction= Non-return rate (PUBMED_ID= 18717969), Non-return rate (PUBMED_ID= 17257192),	MEF2D, RHBG
SC	6	BovineHD0600009601	32965679	Health= Somatic cell score (PUBMED_ID= 15514072), Abomasum displacement (PUBMED_ID= 18946144), Clinical mastitis (PUBMED_ID= 11845286), Bovine spongiform encephalopathy (PUBMED_ID= 15342524), Meat_and_Carcass= Kidney, pelvic, and heart fat percentage (PUBMED_ID= 20477785), Kidney, pelvic, and heart fat weight (PUBMED_ID= 20477785), Kidney fat weight (PUBMED_ID= 20477785), Fat thickness at the 12th rib (PUBMED_ID= 20477797), Marbling score (PUBMED_ID= 20477797), Longissimus muscle area (PUBMED_ID= 20477797), Kidney, pelvic, and heart fat percentage (PUBMED_ID= 18791160), Hematin pigment concentration (PUBMED_ID= 18254735), Carcass weight (PUBMED_ID= 19653884), Milk= Milk fat yield (PUBMED_ID= 11167525), Milk protein percentage (PUBMED_ID= 15514072), Milk solids (PUBMED_ID= 22058003), Milk protein percentage (PUBMED_ID= ISU0040), Milk yield (PUBMED_ID= 19603057), Milk protein yield (PUBMED_ID= 19603057), Milk protein	

				percentage (PUBMED_ID= 8978065), Milk protein percentage (PUBMED_ID= 16702292), Milk protein yield (PUBMED_ID= 16702292), Milk yield (PUBMED_ID= 16702292), Milk yield (PUBMED_ID= 12778594), Milk protein percentage (PUBMED_ID= 12778594), Milk fat percentage (PUBMED_ID= 9691050), Milk fat percentage (PUBMED_ID= 11178740), Milk yield (PUBMED_ID= 11167525), Milk fat yield (PUBMED_ID= 16428646), Milk fat percentage (PUBMED_ID= 16428646), Milk protein percentage (PUBMED_ID= ISU0040), Milk fat yield (PUBMED_ID= 19603057), Milk fat percentage (PUBMED_ID= 19603057), Milk protein percentage (PUBMED_ID= 22742505), Production= Body weight (weaning) (PUBMED_ID= 20477797), Body weight (slaughter) (PUBMED_ID= 19653884), Body weight (birth) (PUBMED_ID= 19016677), Body weight (birth) (PUBMED_ID= 10764062), Body weight (weaning) (PUBMED_ID= 20477797), Body length (birth) (PUBMED_ID= 18791160), Body weight (birth) (PUBMED_ID= 18791160), Body weight (birth) (PUBMED_ID= 15537758), Average daily gain (PUBMED_ID= 15537758), Hip height (PUBMED_ID= 12605852), Rump width (PUBMED_ID= 12605852), Reproduction= Calving ease (PUBMED_ID= 20477797), Gestation length (PUBMED_ID= 19016677),	
SC	8	HAPLOTYPE	38363220-38366097	Exterior=Rump angle (PUBMED_ID= 21831322), Meat_and_Carcass=Fat thickness at the 12th rib (PUBMED_ID= 11325189), Carcass weight (PUBMED_ID= 20477797), Milk=Milking speed (PUBMED_ID= 12605852), Lactation persistency (PUBMED_ID= 19646150), Milk fat yield (PUBMED_ID= 21831322), Milk yield (PUBMED_ID= 21831322), Milk protein yield (PUBMED_ID= 21831322), Production=Body length (birth), (PUBMED_ID= 18791160), Body weight (mature), (PUBMED_ID= 20477797), Body weight (birth), (PUBMED_ID= 20477797), Net merit (PUBMED_ID= 21831322), Body weight (yearling), (PUBMED_ID= 19966163), Reproduction=Stillbirth (PUBMED_ID= 12613879),	UHRF2
SC	9	HAPLOTYPE	15900318-15919300	Health=Clinical mastitis (PUBMED_ID= 18832229), Meat_and_Carcass=Longissimus muscle area (PUBMED_ID= 20477797), Marbling score (PUBMED_ID= 17347893), Milk=Milk fat yield (PUBMED_ID= 11167525), Production=Length of productive life (PUBMED_ID= 18650300), Reproduction=Conception rate (PUBMED_ID= 31299913),	
SC	10	ARS-BFGL-NGS-74837	18541772	Exterior= Angularity (PUBMED_ID= 10791796), Meat_and_Carcass= Fat percentage (PUBMED_ID= 18791160), Marbling score (PUBMED_ID= 14677852), Carcass weight (PUBMED_ID= 14677852), cis-Vaccenic acid content (PUBMED_ID= 20416790), Milk= Milk protein yield (PUBMED_ID= 18298934), Milk fat yield (PUBMED_ID= 7713441), Production= Veterinary treatments (PUBMED_ID= 19389971), Height (mature) (PUBMED_ID= 20477797), Body weight (birth) (PUBMED_ID= 20477797), Reproduction= Conception rate (PUBMED_ID= 31299913), First service conception (PUBMED_ID= 31299913),	THSD4
SC	10	BovineHD1000007178	22308257	Exterior= Social separation--Vocalization (PUBMED_ID= 18784067), Udder attachment (PUBMED_ID= 15377635), Teat length (PUBMED_ID= 15377635), Meat_and_Carcass= Marbling score (PUBMED_ID= 14677852), Carcass weight (PUBMED_ID= 14677852), Longissimus muscle area (PUBMED_ID= 20477797), Muscle pH (PUBMED_ID= 18254735), Muscle nitrogen content (PUBMED_ID= 18254735), Gastrointestinal tract weight (PUBMED_ID= 20477785), Carcass weight (PUBMED_ID= 17347893), Milk= Milk protein yield (PUBMED_ID= 18298934), Milk fat yield (PUBMED_ID= 7713441), Milk yield (PUBMED_ID= 18700999), Milk fat yield (PUBMED_ID= 15377635), Milk protein yield (PUBMED_ID= 15377635), Production= Height (mature) (PUBMED_ID= 20477797), Body weight (birth) (PUBMED_ID= 20477797), Body weight (weaning) (PUBMED_ID= 20477797), Body weight (birth) (PUBMED_ID= 32053968), Reproduction= Calving ease (PUBMED_ID= 20477797), Scrotal circumference (PUBMED_ID= 30997484), Sexual precocity (PUBMED_ID= 30053002),	
SC	13	BovineHD1300021455	73602949	Exterior= Teat length (PUBMED_ID= 14691316), Udder attachment (PUBMED_ID= 16230715), Udder height (PUBMED_ID= 16230715), Udder width (PUBMED_ID= 16230715), Udder depth (PUBMED_ID= 16230715), Udder composite index (PUBMED_ID= 16230715), Meat_and_Carcass= Marbling score (PUBMED_ID= 20477797), Milk= Milk yield (PUBMED_ID= 14762090), Milk protein yield (PUBMED_ID= 14762090), Production= PTA type (PUBMED_ID= 16230715), Body weight (yearling) (PUBMED_ID= 12926775),	MATN4, RBPJL, WFDC15B, MATN4, RBPJL, WFDC15B
SC	13	HAPLOTYPE	73602949-73620495	Exterior=Teat length (PUBMED_ID= 14691316), Udder attachment (PUBMED_ID= 16230715), Udder height (PUBMED_ID= 16230715), Udder width (PUBMED_ID= 16230715), Udder depth (PUBMED_ID= 16230715), Udder composite index (PUBMED_ID= 16230715), Meat_and_Carcass=Marbling score (PUBMED_ID= 20477797), Milk=Milk yield (PUBMED_ID= 14762090), Milk protein yield (PUBMED_ID= 14762090), Production=PTA type (PUBMED_ID= 16230715), Body weight (yearling), (PUBMED_ID= 12926775),	
SC	17	BovineHD1700002151	7548169	Exterior= Rump angle (PUBMED_ID= 12605852), Health= PCVI minus PCVF (PUBMED_ID= 12805560), PCVF minus PCVM (PUBMED_ID= 12805560), PCV variance (PUBMED_ID= 12805560), Final packed red blood cell volume (PUBMED_ID= 12805560), Percentage decrease in PCV up to day 150 after challenge (PUBMED_ID= 12805560), Percentage decrease in PCV up to day 100	DCLK2, LRBA

				after challenge (PUBMED_ID= 12805560), Immunoglobulin G level (PUBMED_ID= 22438944), Meat_and_Carcass= Longissimus muscle area (PUBMED_ID= 20477797), Marbling score (PUBMED_ID= 10764062), Carcass weight (PUBMED_ID= 19937580), Fat thickness at the 12th rib (PUBMED_ID= 19937580), Milk= Milk alpha-casein percentage (PUBMED_ID= 19397519), Milking speed (PUBMED_ID= 29705414), Production= Average daily gain (PUBMED_ID= 17596127), Body weight (birth) (PUBMED_ID= 20477797), Residual feed intake (PUBMED_ID= 17709790),	
SC	17	BovineHD1700004678	15972230	Exterior= Rump angle (PUBMED_ID= 12605852), Health= PCVI minus PCVF (PUBMED_ID= 12805560), PCVF minus PCVM (PUBMED_ID= 12805560), PCV variance (PUBMED_ID= 12805560), Final packed red blood cell volume (PUBMED_ID= 12805560), Percentage decrease in PCV up to day 150 after challenge (PUBMED_ID= 12805560), Percentage decrease in PCV up to day 100 after challenge (PUBMED_ID= 12805560), Meat_and_Carcass= Longissimus muscle area (PUBMED_ID= 20477797), Marbling score (PUBMED_ID= 10764062), Carcass weight (PUBMED_ID= 20477797), Milk= Milk alpha-casein percentage (PUBMED_ID= 19397519), Production= Average daily gain (PUBMED_ID= 17596127), Body weight (yearling) (PUBMED_ID= 20477797),	
SC	19	HAPLOTYPE	43058602-43162697	Exterior=Rump angle (PUBMED_ID= 12605852), Social separation--Standing alert (PUBMED_ID= 18784067), Social separation--Vocalization (PUBMED_ID= 18784067), Teat length (PUBMED_ID= 16230715), Health=Somatic cell score (PUBMED_ID= 15514072), Gastrointestinal nematode burden (PUBMED_ID= 19254385), Meat_and_Carcass=Oleic acid content (PUBMED_ID= 17242864), Stearic acid content (PUBMED_ID= 20477785), Myristic acid content (PUBMED_ID= 17242864), Marbling score (PUBMED_ID= 20477797), Longissimus muscle area (PUBMED_ID= 20477797), Muscle pH (PUBMED_ID= 18254735), Subcutaneous fat thickness (PUBMED_ID= 9720178), Intramuscular fat (PUBMED_ID= 9720178), Myristic acid content (PUBMED_ID= 27112906), Oleic acid content (PUBMED_ID= 27112906), Myristic acid content (PUBMED_ID= 23607548), Oleic acid content (PUBMED_ID= 27112906), Myristic acid content (PUBMED_ID= 27112906), Oleic acid content (PUBMED_ID= 27112906), Marbling score (PUBMED_ID= 33101375), Milk=Milk fat percentage (PUBMED_ID= 15514072), Milk protein percentage (PUBMED_ID= 15514072), Milk stearic acid content (PUBMED_ID= 17242864), Milk trans-vaccenic acid content (PUBMED_ID= 17242864), Milk conjugated linoleic acid content (PUBMED_ID= 17242864), Milk oleic acid content (PUBMED_ID= 17242864), Milk myristic acid content (PUBMED_ID= 17242864), Milk linoleic acid content (PUBMED_ID= 17242864), Milk caproic acid content (PUBMED_ID= 17242864), Milk fat yield (PUBMED_ID= 12605852), Milk caprylic acid content (PUBMED_ID= 17242864), Milk capric acid content (PUBMED_ID= 17242864), Milk lauric acid content (PUBMED_ID= 17242864), Milk fat percentage (PUBMED_ID= 12778594), Production=Body weight (mature), (PUBMED_ID= 20477797), Height (yearling), (PUBMED_ID= 20477797), Body weight (weaning), (PUBMED_ID= 20477797), Body weight (birth), (PUBMED_ID= 20477797), Residual feed intake (PUBMED_ID= 17709790), Reproduction=Ovulation rate (PUBMED_ID= 10656928), Dystocia (PUBMED_ID= 19912419), Scrotal circumference (PUBMED_ID= 20477797),	AARSD1, BRCA1, IFI35, NBR1, RND2, RPL27, RUNDC1, TMEM106A, U2, VAT1
SC	23	BovineHD2300006211	23730493	Exterior= Teat placement - front (PUBMED_ID= 16230715), Health= Parasite detection rate (PUBMED_ID= 12805560), Immunoglobulin G level (PUBMED_ID= 21138580), Meat_and_Carcass= Carcass weight (PUBMED_ID= 20477797), Marbling score (PUBMED_ID= 20477797), Milk= Milk fat yield (PUBMED_ID= 15514072), Milk protein yield (PUBMED_ID= 15514072), Milk yield (PUBMED_ID= 15514072), Milk protein percentage (PUBMED_ID= 10430670), Milk protein percentage (PUBMED_ID= 12778594), Milking speed (PUBMED_ID= 10791796), Milk yield (PUBMED_ID= 12729552), Milking speed (PUBMED_ID= 10430670), Production= Body weight (weaning) (PUBMED_ID= 20477797), Body weight (yearling) (PUBMED_ID= 20477797), Body weight (birth) (PUBMED_ID= 20477797), Body weight (birth) (PUBMED_ID= 15537758), Average daily gain (PUBMED_ID= 15537758), Residual feed intake (PUBMED_ID= 18791150), Reproduction= Percentage live sperm after thawing (PUBMED_ID= 19630877), Twinning (PUBMED_ID= 11003703), Twinning (PUBMED_ID= 15147392), Scrotal circumference (PUBMED_ID= 20477797),	
SC	23	HAPLOTYPE	23730493-23745977	Exterior=Teat placement - front (PUBMED_ID= 16230715), Health=Parasite detection rate (PUBMED_ID= 12805560), Immunoglobulin G level (PUBMED_ID= 21138580), Meat_and_Carcass=Carcass weight (PUBMED_ID= 20477797), Marbling score (PUBMED_ID= 20477797), Milk=Milk fat yield (PUBMED_ID= 15514072), Milk protein yield (PUBMED_ID= 15514072), Milk yield (PUBMED_ID= 15514072), Milk protein percentage (PUBMED_ID= 12778594), Milking speed (PUBMED_ID= 10791796), Milk yield (PUBMED_ID= 12729552), Milking speed (PUBMED_ID= 10430670), Production=Body weight (weaning), (PUBMED_ID= 20477797), Body weight (yearling), (PUBMED_ID= 20477797), Body weight (birth), (PUBMED_ID= 15537758), Average daily gain (PUBMED_ID= 15537758), Residual feed intake (PUBMED_ID= 18791150), Reproduction=Percentage live sperm after thawing (PUBMED_ID= 19630877), Twinning (PUBMED_ID= 15147392), Scrotal circumference (PUBMED_ID= 20477797),	
SC	25	HAPLOTYPE	27577974-27654242	Exterior=Udder attachment (PUBMED_ID= 10791796), Flight from feeder (PUBMED_ID= 18784067), Social separation--Vocalization (PUBMED_ID= 18784067), Health=Gastrointestinal nematode burden (PUBMED_ID= 19254385), Immunoglobulin G level (PUBMED_ID= 21138580), Bovine tuberculosis susceptibility (PUBMED_ID= 26960806), Bovine respiratory disease	AHSP, MRPS17, NIPSNAP2,

				susceptibility (PUBMED_ID= 30229962), Meat_and_Carcass=Carcass weight (PUBMED_ID= 20477797), Tenderness score (PUBMED_ID= 18254735), Milk=Milk yield (PUBMED_ID= 18650300), Production=Body weight (yearling), (PUBMED_ID= 20477797), Body weight (weaning), (PUBMED_ID= 20477797), Reproduction=Calving ease (PUBMED_ID= 20477797), Sperm average path velocity (PUBMED_ID= 19630877),	OR7A153, OR7A53, PSPH, RUSF1, SEPTIN14, ZNF713
FS	1	HAPLOTYPE	60895445-60899823	Health=Initial packed red blood cell volume (PUBMED_ID= 12805560), Meat_and_Carcass=Oleic acid content (PUBMED_ID= 20477785), Linoleic acid content (PUBMED_ID= 20416790), Linolenic acid content (PUBMED_ID= 20416790), Polyunsaturated fatty acid content (PUBMED_ID= 20416790), Marbling score (PUBMED_ID= 20477797), Fat percentage (PUBMED_ID= 14731222), Retail product yield (PUBMED_ID= 14731222), Yield grade (PUBMED_ID= 14731222), Marbling score (PUBMED_ID= 33101375), Milk=Milk protein yield (PUBMED_ID= 11178740), Milk yield (PUBMED_ID= 11178740), Milk alpha-casein percentage (PUBMED_ID= 19397519), Production=Body weight (mature), (PUBMED_ID= 20477797), Reproduction=Fertility treatments (PUBMED_ID= 19841231), Conception rate (PUBMED_ID= 12605852),	
FS	2	ARS-BFGL-NGS-60458	28349857	Exterior= Udder depth (PUBMED_ID= 17433017), Body form composite index (PUBMED_ID= 16230715), Teat placement (PUBMED_ID= 12605852), Meat_and_Carcass= Lung percentage (PUBMED_ID= 20477785), Fat thickness at the 12th rib (PUBMED_ID= 14677852), Milk= Milk fat yield (PUBMED_ID= 17433017), Milk fat yield (PUBMED_ID= 16167984), Milk protein percentage (PUBMED_ID= 14762090), Milk yield (PUBMED_ID= 12778594), Production= Body depth (PUBMED_ID= 16230715),	
FS	2	BTB-01145846	56674297	Exterior= Udder depth (PUBMED_ID= 17433017), Health= Immunoglobulin G level (PUBMED_ID= 21138580), Initial packed red blood cell volume (PUBMED_ID= 12805560), PCVI minus PCVF (PUBMED_ID= 12805560), PCVI minus PCVM (PUBMED_ID= 12805560), Minimum packed red blood cell volume (PUBMED_ID= 12805560), Percentage decrease in PCV up to day 150 after challenge (PUBMED_ID= 12805560), Percentage decrease in PCV up to day 100 after challenge (PUBMED_ID= 12805560), Percentage decrease in body weight up to day 150 after challenge (PUBMED_ID= 12805560), Tick resistance (PUBMED_ID= 20433753), Meat_and_Carcass= Lung percentage (PUBMED_ID= 20477785), Fat thickness at the 12th rib (PUBMED_ID= 14677852), Yield grade (PUBMED_ID= 14677852), Carcass weight (PUBMED_ID= 20477797), Fat thickness at the 12th rib (PUBMED_ID= 20477797), Milk= Milk fat yield (PUBMED_ID= 17433017), Milk yield (PUBMED_ID= 12778594), Milk fat percentage (PUBMED_ID= 14762090), Milk fat yield (PUBMED_ID= 9691050), Production= Body weight (birth) (PUBMED_ID= 14677852), Body weight (initial) (PUBMED_ID= 12805560), Reproduction= Non-return rate (PUBMED_ID= 18717969),	LRP1B
FS	2	HAPLOTYPE	28329020-28349857	Exterior=Udder depth (PUBMED_ID= 17433017), Body form composite index (PUBMED_ID= 16230715), Teat placement (PUBMED_ID= 12605852), Meat_and_Carcass=Lung percentage (PUBMED_ID= 20477785), Fat thickness at the 12th rib (PUBMED_ID= 14677852), Milk=Milk fat yield (PUBMED_ID= 16167984), Milk protein percentage (PUBMED_ID= 14762090), Milk yield (PUBMED_ID= 12778594), Production=Body depth (PUBMED_ID= 16230715),	
FS	3	BovineHD0300029985	104074474	Exterior= Structural soundness (PUBMED_ID= 17183116), Health= Clinical mastitis (PUBMED_ID= 11845286), Meat_and_Carcass= Carcass weight (PUBMED_ID= 20477797), Marbling score (PUBMED_ID= 20477797), Reproduction= Stillbirth (PUBMED_ID= 18420641),	
FS	3	HAPLOTYPE	104074474-104081372	Exterior=Structural soundness (PUBMED_ID= 17183116), Health=Clinical mastitis (PUBMED_ID= 11845286), Meat_and_Carcass=Carcass weight (PUBMED_ID= 20477797), Marbling score (PUBMED_ID= 20477797), Reproduction=Stillbirth (PUBMED_ID= 18420641),	
FS	4	BTB-00190917	59503146	Exterior= Social separation--Vocalization (PUBMED_ID= 18784067), Social separation--Standing alert (PUBMED_ID= 18784067), Health= FMDV peptide-induced cell proliferation (PUBMED_ID= 21138580), Meat_and_Carcass= Marbling score (PUBMED_ID= 19781039), Longissimus muscle area (PUBMED_ID= 15537759), Marbling score (PUBMED_ID= 15537759), Milk= Milking speed (PUBMED_ID= 15377635), Milk protein yield (PUBMED_ID= 15377635), Milk fat percentage (PUBMED_ID= 9621249), Milk caproic acid content (PUBMED_ID= 34091779), Production= Body weight (weaning) (PUBMED_ID= 20477797), Body weight (slaughter) (PUBMED_ID= 22303340), Reproduction= Calving ease (PUBMED_ID= 18420641),	
FS	4	HAPLOTYPE	59489126-59503146	Exterior=Social separation--Vocalization (PUBMED_ID= 18784067), Social separation--Standing alert (PUBMED_ID= 18784067), Health=FMDV peptide-induced cell proliferation (PUBMED_ID= 21138580), Meat_and_Carcass=Marbling score (PUBMED_ID= 19781039), Longissimus muscle area (PUBMED_ID= 15537759), Marbling score (PUBMED_ID= 15537759), Milk=Milking speed (PUBMED_ID= 15377635), Milk protein yield (PUBMED_ID= 15377635), Milk fat percentage (PUBMED_ID= 9621249), Milk caproic	

				acid content (PUBMED_ID= 34091779), Production=Body weight (weaning), (PUBMED_ID= 20477797), Body weight (slaughter), (PUBMED_ID= 22303340), Reproduction=Calving ease (PUBMED_ID= 18420641),	
FS	4	HAPLOTYPE	6351817-6418053	Meat_and_Carcass=Tenderness score (PUBMED_ID= 33101375), Production=Body depth (PUBMED_ID= 16230715), Body weight gain (PUBMED_ID= 19966163), Reproduction=Inseminations per conception (PUBMED_ID= 32650431),	
FS	5	BovineHD0500035146	118776285	Meat_and_Carcass= Carcass weight (PUBMED_ID= 20477797), Reproduction= Scrotal circumference (PUBMED_ID= 20477797),	
FS	6	BovineHD4100004588	37588140	Health= Somatic cell score (PUBMED_ID= 15514072), Clinical mastitis (PUBMED_ID= 11845286), Bovine spongiform encephalopathy (PUBMED_ID= 15342524), Meat_and_Carcass= Kidney, pelvic, and heart fat percentage (PUBMED_ID= 20477785), Kidney, pelvic, and heart fat weight (PUBMED_ID= 20477785), Kidney fat weight (PUBMED_ID= 20477785), Fat thickness at the 12th rib (PUBMED_ID= 20477797), Marbling score (PUBMED_ID= 20477797), Longissimus muscle area (PUBMED_ID= 20477797), Kidney, pelvic, and heart fat percentage (PUBMED_ID= 18791160), Hematin pigment concentration (PUBMED_ID= 18254735), Carcass weight (PUBMED_ID= 19653884), Longissimus muscle area (PUBMED_ID= 31931697), Subcutaneous fat thickness (PUBMED_ID= 31931697), Carcass weight (PUBMED_ID= 31931697), Lean meat yield (PUBMED_ID= 31931697), Subcutaneous fat thickness (PUBMED_ID= 31931697), Longissimus muscle area (PUBMED_ID= 31931697), Carcass weight (PUBMED_ID= 31931697), Bone weight (PUBMED_ID= 34912371), Fat thickness at the 12th rib (PUBMED_ID= 22168586), Carcass weight (PUBMED_ID= 22168586), Longissimus muscle area (PUBMED_ID= 22168586), Carcass weight (PUBMED_ID= 31931697), Longissimus muscle area (PUBMED_ID= 31931697), Bone weight (PUBMED_ID= 34912371), Carcass weight (PUBMED_ID= 31931697), Longissimus muscle area (PUBMED_ID= 31931697), Carcass weight (PUBMED_ID= 31931697), Bone weight (PUBMED_ID= 34912371), Carcass weight (PUBMED_ID= 31931697), Bone weight (PUBMED_ID= 34912371), Carcass weight (PUBMED_ID= 31931697), Longissimus muscle area (PUBMED_ID= 31931697), Bone weight (PUBMED_ID= 34912371), Longissimus muscle area (PUBMED_ID= 31931697), Subcutaneous fat thickness (PUBMED_ID= 31931697), Lean meat yield (PUBMED_ID= 31931697), Carcass weight (PUBMED_ID= 31931697), Bone weight (PUBMED_ID= 34912371), Carcass weight (PUBMED_ID= 31931697), Longissimus muscle area (PUBMED_ID= 31931697), Subcutaneous fat thickness (PUBMED_ID= 31931697), Lean meat yield (PUBMED_ID= 31931697), Carcass weight (PUBMED_ID= 31931697), Bone weight (PUBMED_ID= 34912371), Carcass weight (PUBMED_ID= 31931697), Longissimus muscle area (PUBMED_ID= 31931697), Subcutaneous fat thickness (PUBMED_ID= 31931697), Lean meat yield (PUBMED_ID= 31931697), Carcass weight (PUBMED_ID= 31931697), Bone weight (PUBMED_ID= 34912371), Carcass weight (PUBMED_ID= 31931697), Bone weight (PUBMED_ID= 34912371), Carcass weight (PUBMED_ID= 31931697), Longissimus muscle area (PUBMED_ID= 31931697), Carcass weight (PUBMED_ID= 31931697), Longissimus muscle area (PUBMED_ID= 31931697), Bone weight (PUBMED_ID= 34912371), Carcass weight (PUBMED_ID= 31931697), Subcutaneous fat thickness (PUBMED_ID= 31931697), Longissimus muscle area (PUBMED_ID= 31931697), Carcass weight (PUBMED_ID= 31931697), Lean meat yield (PUBMED_ID= 31931697), Bone weight (PUBMED_ID= 34912371), Carcass weight (PUBMED_ID= 31931697), Subcutaneous fat thickness (PUBMED_ID= 31931697), Longissimus muscle area (PUBMED_ID= 31931697), Lean meat yield (PUBMED_ID= 31931697), Carcass weight (PUBMED_ID= 31931697), Bone weight (PUBMED_ID= 34912371), Carcass weight (PUBMED_ID= 31931697), Longissimus muscle area (PUBMED_ID= 31931697), Bone weight (PUBMED_ID= 34912371), Longissimus muscle area (PUBMED_ID= 31931697), Subcutaneous fat thickness (PUBMED_ID= 31931697), Carcass weight (PUBMED_ID= 31931697), Lean meat yield (PUBMED_ID= 31931697), Subcutaneous fat thickness (PUBMED_ID= 31931697), Longissimus muscle area (PUBMED_ID= 31931697), Carcass weight (PUBMED_ID= 31931697), Bone weight (PUBMED_ID= 34912371), Carcass weight (PUBMED_ID= 31931697), Lean meat yield (PUBMED_ID= 31931697), Subcutaneous fat thickness (PUBMED_ID= 31931697), Longissimus muscle area (PUBMED_ID= 31931697), Bone weight (PUBMED_ID= 34912371), Milk= Milk fat yield (PUBMED_ID= 11167525), Milk protein percentage (PUBMED_ID= 15514072), Milk solids (PUBMED_ID= 22058003), Milk protein percentage (PUBMED_ID= ISU0040), Milk yield (PUBMED_ID= 19603057), Milk protein yield (PUBMED_ID= 19603057), Milk protein percentage (PUBMED_ID= 8978065), Milk protein percentage (PUBMED_ID= 16702292), Milk protein yield (PUBMED_ID= 16702292), Milk yield (PUBMED_ID= 16702292), Milk yield (PUBMED_ID= 12778594), Milk protein percentage (PUBMED_ID= 12778594), Milk fat percentage (PUBMED_ID= 9691050), Milk fat percentage (PUBMED_ID= 11178740), Milk yield (PUBMED_ID= 11167525), Milk fat yield (PUBMED_ID= 16428646), Milk fat percentage (PUBMED_ID= 16428646), Milk protein percentage (PUBMED_ID= ISU0040), Milk yield (PUBMED_ID= 16428646), Milk protein percentage (PUBMED_ID= 19397519), Milk protein yield (PUBMED_ID= 16428646), Milk yield (PUBMED_ID= ISU0040), Milk protein percentage (PUBMED_ID= 27760518), Milk fat percentage (PUBMED_ID= 27760518), Milk protein percentage (PUBMED_ID= 27760518), Milk fat percentage (PUBMED_ID= 27760518), Milk protein percentage (PUBMED_ID= 27760518), Milk fat percentage (PUBMED_ID= 32635893), Milk protein percentage (PUBMED_ID= 27760518), Milk fat percentage (PUBMED_ID=	LCORL

				27760518), Milk protein percentage (PUBMED_ID= 27760518), Production= Body weight (weaning) (PUBMED_ID= 20477797), Body weight (slaughter) (PUBMED_ID= 19653884), Body weight (birth) (PUBMED_ID= 19016677), Body weight (birth) (PUBMED_ID= 10764062), Body weight (weaning) (PUBMED_ID= 20477797), Body length (birth) (PUBMED_ID= 18791160), Body weight (birth) (PUBMED_ID= 18791160), Body weight (birth) (PUBMED_ID= 15537758), Average daily gain (PUBMED_ID= 15537758), Hip height (PUBMED_ID= 12605852), Rump width (PUBMED_ID= 12605852), Metabolic body weight (PUBMED_ID= 31931702), Average daily gain (PUBMED_ID= 31931702), Dry matter intake (PUBMED_ID= 31931702), Average daily gain (PUBMED_ID= 31931702), Metabolic body weight (PUBMED_ID= 31931702), Dry matter intake (PUBMED_ID= 31931702), Average daily gain (PUBMED_ID= 31931702), Average daily feed intake (PUBMED_ID= 22168586), Average daily gain (PUBMED_ID= 22168586), Metabolic body weight (PUBMED_ID= 31931702), Average daily gain (PUBMED_ID= 31931702), Dry matter intake (PUBMED_ID= 31931702), Metabolic body weight (PUBMED_ID= 31931702), Dry matter intake (PUBMED_ID= 31931702), Metabolic body weight (PUBMED_ID= 31931702), Average daily gain (PUBMED_ID= 31931702), Metabolic body weight (PUBMED_ID= 31931702), Average daily gain (PUBMED_ID= 31931702), Dry matter intake (PUBMED_ID= 31931702), Metabolic body weight (PUBMED_ID= 31931702), Average daily gain (PUBMED_ID= 31931702), Metabolic body weight (PUBMED_ID= 31931702), Average daily gain (PUBMED_ID= 31931702), Metabolic body weight (PUBMED_ID= 31931702), Average daily gain (PUBMED_ID= 31931702), Dry matter intake (PUBMED_ID= 31931702), Metabolic body weight (PUBMED_ID= 31931702), Average daily gain (PUBMED_ID= 31931702), Metabolic body weight (PUBMED_ID= 31931702), Average daily gain (PUBMED_ID= 30290764), Body weight (birth) (PUBMED_ID= 30290764), Average daily gain (PUBMED_ID= 30290764), Body weight (birth) (PUBMED_ID= 30290764), Body weight (birth) (PUBMED_ID= 19966163), Body weight (weaning) (PUBMED_ID= 19966163), Body weight (yearling) (PUBMED_ID= 19966163), Body weight gain (PUBMED_ID= 19966163), Metabolic body weight (PUBMED_ID= 31931702), Average daily gain (PUBMED_ID= 31931702), Dry matter intake (PUBMED_ID= 31931702), Metabolic body weight (PUBMED_ID= 31931702), Dry matter intake (PUBMED_ID= 31931702), Average daily gain (PUBMED_ID= 31931702), Metabolic body weight (PUBMED_ID= 31931702), Dry matter intake (PUBMED_ID= 31931702), Average daily gain (PUBMED_ID= 31931702), Metabolic body weight (PUBMED_ID= 31931702), Dry matter intake (PUBMED_ID= 31931702), Average daily gain (PUBMED_ID= 31931702), Metabolic body weight (PUBMED_ID= 31931702), Average daily gain (PUBMED_ID= 31931702), Metabolic body weight (PUBMED_ID= 31931702), Dry matter intake (PUBMED_ID= 31931702), Metabolic body weight (PUBMED_ID= 31931702), Average daily gain (PUBMED_ID= 31931702), Metabolic body weight (PUBMED_ID= 31931702), Dry matter intake (PUBMED_ID= 31931702), Metabolic body weight (PUBMED_ID= 31931702), Body weight (birth) (PUBMED_ID= 23851991), Body weight (weaning) (PUBMED_ID= 23851991), Body weight (birth) (PUBMED_ID= 19966163), Body weight gain (PUBMED_ID= 19966163), Metabolic body weight (PUBMED_ID= 31931702), Dry matter intake (PUBMED_ID= 31931702), Metabolic body weight (PUBMED_ID= 31931702), Average daily gain (PUBMED_ID= 31931702), Reproduction= Calving ease (PUBMED_ID= 20477797), Gestation length (PUBMED_ID= 19016677), Calf size (PUBMED_ID= 25935543), Calving index (PUBMED_ID= 25935543),	
FS	7	BovineHD0700000068	467158	Meat_and_Carcass= Marbling score (PUBMED_ID= 20477797), Carcass weight (PUBMED_ID= 17596127), Fat thickness at the 12th rib (PUBMED_ID= 22303340), Longissimus muscle area (PUBMED_ID= 32180796), Milk= Milk beta-casein percentage (PUBMED_ID= 19397519), Milk fat yield (PUBMED_ID= 14762091), Milk fat percentage (PUBMED_ID= 14762091), Production= Body weight (weaning) (PUBMED_ID= 20477797), Reproduction= Scrotal circumference (PUBMED_ID= 20477797), Dystocia (PUBMED_ID= 12613879), Stillbirth (PUBMED_ID= 12613879),	5S_rRNA, FLT4
FS	8	BovineHD0800015325	50833954	Health= Clinical mastitis (PUBMED_ID= 11845286), Somatic cell count (PUBMED_ID= 11845286), Meat_and_Carcass= Carcass weight (PUBMED_ID= 20477797), Longissimus muscle area (PUBMED_ID= 20477797), Muscle anserine content (PUBMED_ID= 29163638), Milk= Milking speed (PUBMED_ID= 12605852), Production= Body length (birth) (PUBMED_ID= 18791160), Body weight (birth) (PUBMED_ID= 20477797), Height (yearling) (PUBMED_ID= 20477797), Height (mature) (PUBMED_ID= 20477797), Body weight (yearling) (PUBMED_ID= 19966163), Reproduction= Dystocia (PUBMED_ID= 12613879),	TRPM6
FS	8	BovineHD0800019879	65744992	Exterior= Structural soundness (PUBMED_ID= 16167984), Foot angle (PUBMED_ID= 17183116), Health= Clinical mastitis (PUBMED_ID= 11845286), Somatic cell count (PUBMED_ID= 11845286), Gastrointestinal nematode burden (PUBMED_ID= 24303892), Somatic cell count (PUBMED_ID= 16167984), Milk= Milk kappa-casein percentage (PUBMED_ID= 27485317), Milk unglycosylated kappa-casein percentage (PUBMED_ID= 27485317), Production= Body weight (birth) (PUBMED_ID= 20477797), Body weight (birth) (PUBMED_ID= 19966163), Reproduction= Dystocia (PUBMED_ID= 12613879), Calving ease (PUBMED_ID= 18420641), Stillbirth (PUBMED_ID= 18420641), Age at puberty (PUBMED_ID= 22100599),	
FS	8	BovineHD0800027652	91458487	Exterior= Foot angle (PUBMED_ID= 17183116), Structural soundness (PUBMED_ID= 16167984), Meat_and_Carcass= Carcass weight (PUBMED_ID= 20477797), Marbling score (PUBMED_ID= 20477797), Longissimus muscle area (PUBMED_ID= 20477797),	GRIN3A

				Intramuscular fat (PUBMED_ID= 21421834), Milk= Milk protein yield (PUBMED_ID= 29751743), Milk protein yield (PUBMED_ID= 30696404), Production= Body weight (birth) (PUBMED_ID= 20477797), Height (mature) (PUBMED_ID= 20477797), Reproduction= Calving ease (PUBMED_ID= 18420641), Stillbirth (PUBMED_ID= 18420641), Twinning (PUBMED_ID= 16026340), Calving ease (PUBMED_ID= 20477797),	
FS	8	BovineHD4100007105	102704398	Exterior= Foot angle (PUBMED_ID= 17183116), Structural soundness (PUBMED_ID= 16167984), Meat_and_Carcass= Carcass weight (PUBMED_ID= 20477797), Marbling score (PUBMED_ID= 20477797), Longissimus muscle area (PUBMED_ID= 20477797), Production= Body weight (birth) (PUBMED_ID= 20477797), Height (mature) (PUBMED_ID= 20477797), Rump width (PUBMED_ID= 12605852), Reproduction= Calving ease (PUBMED_ID= 18420641), Stillbirth (PUBMED_ID= 18420641), Twinning (PUBMED_ID= 16026340), Calving ease (PUBMED_ID= 20477797),	C8H9orf43, POLE3, RGS3, SNORA72
FS	8	HAPLOTYPE	65744992-65802549	Exterior=Structural soundness (PUBMED_ID= 16167984), Foot angle (PUBMED_ID= 17183116), Health=Clinical mastitis (PUBMED_ID= 11845286), Somatic cell count (PUBMED_ID= 11845286), Gastrointestinal nematode burden (PUBMED_ID= 24303892), Somatic cell count (PUBMED_ID= 16167984), Milk=Milk kappa-casein percentage (PUBMED_ID= 27485317), Milk unglycosylated kappa-casein percentage (PUBMED_ID= 27485317), Milk kappa-casein percentage (PUBMED_ID= 27485317), Production=Body weight (birth), (PUBMED_ID= 19966163), Reproduction=Dystocia (PUBMED_ID= 12613879), Calving ease (PUBMED_ID= 18420641), Stillbirth (PUBMED_ID= 18420641), Age at puberty (PUBMED_ID= 22100599), Conception rate (PUBMED_ID= 31718557), Inseminations per conception (PUBMED_ID= 31718557),	
FS	11	BovineHD1100027596	94731004	Health= Immunoglobulin G level (PUBMED_ID= 19016677), Meat_and_Carcass= Marbling score (PUBMED_ID= 20477797), Longissimus muscle area (PUBMED_ID= 20477797), Milk= Cheese protein recovery (PUBMED_ID= 27889122), Milk fat yield (PUBMED_ID= 34828436), Cheese protein recovery (PUBMED_ID= 27889122), Production= Body weight (mature) (PUBMED_ID= 20477797), Body weight (weaning) (PUBMED_ID= 20477797),	DENND1A
FS	12	HAPLOTYPE	50678326-50689493	Exterior=Structural soundness (PUBMED_ID= 17183116), Health=Gastrointestinal nematode burden (PUBMED_ID= 24303892), Meat_and_Carcass=Retail product yield (PUBMED_ID= 14677852), Longissimus muscle area (PUBMED_ID= 20477797), Milk=Milk fat yield (PUBMED_ID= 12778594), Production=Body weight (yearling), (PUBMED_ID= 20477797), Height (mature), (PUBMED_ID= 20477797), Body weight (mature), (PUBMED_ID= 20477797), Body weight (birth), (PUBMED_ID= 20477797), Body weight (mature), (PUBMED_ID= 20477797), Body weight (weaning), (PUBMED_ID= 20477797), Reproduction=Stillbirth (PUBMED_ID= 19912419),	LMO7, UCHL3
FS	16	BovineHD1600003330	11855136	Exterior= Structural soundness (PUBMED_ID= 17183116), Meat_and_Carcass= Fat thickness at the 12th rib (PUBMED_ID= 20477797), Carcass weight (PUBMED_ID= 20477797), Milk= 305-day milk yield (PUBMED_ID= 17582132), Milk protein yield (PUBMED_ID= 17582132), Production= Length of productive life (PUBMED_ID= 9691050), Body weight (weaning) (PUBMED_ID= 20477797), Height (mature) (PUBMED_ID= 20477797), Reproduction= Stillbirth (PUBMED_ID= 19912419), Interval to first estrus after calving (PUBMED_ID= 22100599),	
FS	17	ARS-BFGL-NGS-118918	18626728	Exterior= Rump angle (PUBMED_ID= 12605852), Health= PCVI minus PCVF (PUBMED_ID= 12805560), PCVF minus PCVM (PUBMED_ID= 12805560), PCV variance (PUBMED_ID= 12805560), Final packed red blood cell volume (PUBMED_ID= 12805560), Percentage decrease in PCV up to day 150 after challenge (PUBMED_ID= 12805560), Percentage decrease in PCV up to day 100 after challenge (PUBMED_ID= 12805560), Meat_and_Carcass= Marbling score (PUBMED_ID= 10764062), Milk= Milk alpha-casein percentage (PUBMED_ID= 19397519), Milk yield (PUBMED_ID= 21607666), Lactation persistency (PUBMED_ID= 21607666), Milk yield (PUBMED_ID= 21607666), Production= Average daily gain (PUBMED_ID= 17596127), Body weight (weaning) (PUBMED_ID= 20477797), Reproduction= Calving ease (PUBMED_ID= 20477797),	NOCT
FS	17	BTB-01087937	19461407	Exterior= Rump angle (PUBMED_ID= 12605852), Health= PCVI minus PCVF (PUBMED_ID= 12805560), PCVF minus PCVM (PUBMED_ID= 12805560), PCV variance (PUBMED_ID= 12805560), Final packed red blood cell volume (PUBMED_ID= 12805560), Percentage decrease in PCV up to day 150 after challenge (PUBMED_ID= 12805560), Percentage decrease in PCV up to day 100 after challenge (PUBMED_ID= 12805560), Meat_and_Carcass= Marbling score (PUBMED_ID= 10764062), Milk= Milk alpha-casein percentage (PUBMED_ID= 19397519), Production= Average daily gain (PUBMED_ID= 17596127), Body weight (weaning) (PUBMED_ID= 20477797), Reproduction= Calving ease (PUBMED_ID= 20477797), Early embryonic survival (PUBMED_ID= 19456315),	SLC7A11
FS	17	BovineHD1700006112	20844627	Exterior= Rump angle (PUBMED_ID= 12605852), Health= PCVI minus PCVF (PUBMED_ID= 12805560), PCVF minus PCVM (PUBMED_ID= 12805560), PCV variance (PUBMED_ID= 12805560), Final packed red blood cell volume (PUBMED_ID= 12805560), Percentage decrease in PCV up to day 150 after challenge (PUBMED_ID= 12805560), Percentage decrease in PCV up to day 100 after challenge (PUBMED_ID= 12805560), Gastrointestinal nematode burden (PUBMED_ID= 24303892), Meat_and_Carcass=	

				Marbling score (PUBMED_ID= 10764062), Milk= Milk alpha-casein percentage (PUBMED_ID= 19397519), Production= Average daily gain (PUBMED_ID= 17596127), Body weight (weaning) (PUBMED_ID= 20477797), Reproduction= Calving ease (PUBMED_ID= 20477797),	
FS	17	BovineHD1700007095	24825598	Health= PCVI minus PCVF (PUBMED_ID= 12805560), PCVF minus PCVM (PUBMED_ID= 12805560), PCV variance (PUBMED_ID= 12805560), Final packed red blood cell volume (PUBMED_ID= 12805560), Percentage decrease in PCV up to day 150 after challenge (PUBMED_ID= 12805560), Percentage decrease in PCV up to day 100 after challenge (PUBMED_ID= 12805560), Meat_and_Carcass= Marbling score (PUBMED_ID= 10764062), Milk= Milk alpha-casein percentage (PUBMED_ID= 19397519), Production= Average daily gain (PUBMED_ID= 17596127), Body weight (weaning) (PUBMED_ID= 20477797), Reproduction= Calving ease (PUBMED_ID= 20477797),	
FS	18	ARS-BFGL-NGS-103183	47994061	Exterior= Foot angle (PUBMED_ID= 16167984), Teat length (PUBMED_ID= 16167984), Health= Immunoglobulin G level (PUBMED_ID= 21138580), Somatic cell score (PUBMED_ID= 19725965), Bovine respiratory disease susceptibility (PUBMED_ID= 34409086), Bovine respiratory disease susceptibility (PUBMED_ID= ISU0120), Meat_and_Carcass= Palmitic acid content (PUBMED_ID= 20477785), Omega-6 to omega-3 fatty acid ratio (PUBMED_ID= 20416790), Milk= Milk fat yield (PUBMED_ID= 15514072), Milk protein yield (PUBMED_ID= 12487480), Milk yield (PUBMED_ID= 16533362), Production= Body weight (birth) (PUBMED_ID= 20477797), Reproduction= Stillbirth (PUBMED_ID= 18420641),	DPF1, SIPA1L3, U6
FS	19	BovineHD1900017112	59345427	Exterior= Foot angle (PUBMED_ID= 21831322), Feet and leg conformation (PUBMED_ID= 21831322), Teat placement - front (PUBMED_ID= 21831322), Udder attachment (PUBMED_ID= 21831322), Rear leg placement - rear view (PUBMED_ID= 21831322), Teat placement - rear (PUBMED_ID= 21831322), Udder height (PUBMED_ID= 21831322), Stature (PUBMED_ID= 21831322), Strength (PUBMED_ID= 21831322), Udder cleft (PUBMED_ID= 21831322), Udder depth (PUBMED_ID= 21831322), Health= Somatic cell score (PUBMED_ID= 15514072), Abomasum displacement (PUBMED_ID= 18946144), Bovine spongiform encephalopathy (PUBMED_ID= 15342524), Meat_and_Carcass= Myristic acid content (PUBMED_ID= 17242864), Marbling score (PUBMED_ID= 20477797), Subcutaneous fat thickness (PUBMED_ID= 15080315), Longissimus muscle area (PUBMED_ID= 20477797), Milk= Milk protein percentage (PUBMED_ID= 15514072), Milk conjugated linoleic acid content (PUBMED_ID= 17242864), Milk fat yield (PUBMED_ID= 12605852), Milk yield (PUBMED_ID= 22449276), Milk tricosanoic acid content (PUBMED_ID= 27506634), Production= Body weight (birth) (PUBMED_ID= 9720178), Body weight (yearling) (PUBMED_ID= 20477797), Body weight (mature) (PUBMED_ID= 26445451), Body depth (PUBMED_ID= 21831322), PTA type (PUBMED_ID= 21831322), Net merit (PUBMED_ID= 21831322), Rump width (PUBMED_ID= 21831322), Reproduction= Calving ease (PUBMED_ID= 20477797), Scrotal circumference (PUBMED_ID= 20477797), Conception rate (PUBMED_ID= 31718557), Calving ease (maternal) (PUBMED_ID= 21831322), Calving ease (PUBMED_ID= 21831322), Conception rate (PUBMED_ID= 31299913),	
FS	19	BovineHD1900018261	62590391	Health= Bovine spongiform encephalopathy (PUBMED_ID= 15342524), Milk= Milk fat yield (PUBMED_ID= 21831322), Milk yield (PUBMED_ID= 21831322), Milk protein yield (PUBMED_ID= 21831322), Milk protein yield (PUBMED_ID= 22449276),	APOH, CEP112
FS	20	BovineHD2000020384	69702259	Meat_and_Carcass= Marbling score (PUBMED_ID= 20477797), Shear force (PUBMED_ID= 28727016), Milk= Milk protein yield (PUBMED_ID= 18298934), Colostrum albumin concentration (PUBMED_ID= 33255903), Reproduction= Calving ease (PUBMED_ID= 20477797),	
FS	22	Hapmap53119-rs29018	29120539	Health= Minimum packed red blood cell volume (PUBMED_ID= 12805560), Meat_and_Carcass= Intramuscular fat (PUBMED_ID= 18254735), Marbling score (PUBMED_ID= 18791160), Myristic acid content (PUBMED_ID= 20416790), Palmitic acid content (PUBMED_ID= 20416790), Palmitoleic acid content (PUBMED_ID= 20416790), Stearic acid content (PUBMED_ID= 20416790), Oleic acid content (PUBMED_ID= 20416790), Conjugated linoleic acid content (PUBMED_ID= 20416790), Total fatty acid content (PUBMED_ID= 20416790), Saturated fatty acid content (PUBMED_ID= 20416790), Polyunsaturated to saturated fatty acid ratio (PUBMED_ID= 20416790), Carcass weight (PUBMED_ID= 20477797), Milk= Milk protein yield (PUBMED_ID= 14762090), Production= Body weight (yearling) (PUBMED_ID= 20477797), Reproduction= Non-return rate (PUBMED_ID= 19389971),	
FS	22	BovineHD2200009696	33624719	Exterior= Udder depth (PUBMED_ID= 18832229), Health= Minimum packed red blood cell volume (PUBMED_ID= 12805560), Somatic cell score (PUBMED_ID= 18832229), Meat_and_Carcass= Beef flavor intensity (PUBMED_ID= 18254735), Fat percentage (PUBMED_ID= 18791160), Marbling score (PUBMED_ID= 33101375), Production= Body weight (yearling) (PUBMED_ID= 20477797), Height (mature) (PUBMED_ID= 20477797), Reproduction= Calf size (PUBMED_ID= 18420641),	
FS	22	BovineHD2200009726	33760924	Exterior= Udder depth (PUBMED_ID= 18832229), Rump angle (PUBMED_ID= 16230715), Health= Minimum packed red blood cell volume (PUBMED_ID= 12805560), Somatic cell score (PUBMED_ID= 18832229), Meat_and_Carcass= Beef flavor intensity (PUBMED_ID= 18254735), Fat percentage (PUBMED_ID= 18791160), Production= Body weight (yearling) (PUBMED_ID= 20477797), Height (mature) (PUBMED_ID= 20477797), Reproduction= Calf size (PUBMED_ID= 18420641),	

HF	2	HAPLOTYPE	44282846-44313656	Exterior=Udder depth (PUBMED_ID= 17433017), Teat placement (PUBMED_ID= 12605852), Dairy form (PUBMED_ID= 16167984), Health=Immunoglobulin G level (PUBMED_ID= 21138580), Initial packed red blood cell volume (PUBMED_ID= 12805560), PCVI minus PCVF (PUBMED_ID= 12805560), PCVI minus PCVM (PUBMED_ID= 12805560), Minimum packed red blood cell volume (PUBMED_ID= 12805560), Percentage decrease in PCV up to day 150 after challenge (PUBMED_ID= 12805560), Percentage decrease in PCV up to day 100 after challenge (PUBMED_ID= 12805560), Percentage decrease in body weight up to day 150 after challenge (PUBMED_ID= 12805560), Meat_and_Carcass=Lung percentage (PUBMED_ID= 20477785), Fat thickness at the 12th rib (PUBMED_ID= 14677852), Yield grade (PUBMED_ID= 14677852), Milk=Milk fat yield (PUBMED_ID= 17433017), Milk yield (PUBMED_ID= 12778594), Milk protein yield (PUBMED_ID= 16167984), Milk fat percentage (PUBMED_ID= 14762090), Milk fat yield (PUBMED_ID= 9691050), Production=Body weight (birth), (PUBMED_ID= 14677852), Chest depth (PUBMED_ID= 12605852), Body weight (birth), (PUBMED_ID= 20477797), Body weight (initial), (PUBMED_ID= 12805560),	ARL5A, CACNB4
HF	2	HAPLOTYPE	44292504-44317544	Exterior=Udder depth (PUBMED_ID= 17433017), Teat placement (PUBMED_ID= 12605852), Dairy form (PUBMED_ID= 16167984), Health=Immunoglobulin G level (PUBMED_ID= 21138580), Initial packed red blood cell volume (PUBMED_ID= 12805560), PCVI minus PCVF (PUBMED_ID= 12805560), PCVI minus PCVM (PUBMED_ID= 12805560), Minimum packed red blood cell volume (PUBMED_ID= 12805560), Percentage decrease in PCV up to day 150 after challenge (PUBMED_ID= 12805560), Percentage decrease in PCV up to day 100 after challenge (PUBMED_ID= 12805560), Percentage decrease in body weight up to day 150 after challenge (PUBMED_ID= 12805560), Meat_and_Carcass=Lung percentage (PUBMED_ID= 20477785), Fat thickness at the 12th rib (PUBMED_ID= 14677852), Yield grade (PUBMED_ID= 14677852), Milk=Milk fat yield (PUBMED_ID= 17433017), Milk yield (PUBMED_ID= 12778594), Milk protein yield (PUBMED_ID= 16167984), Milk fat percentage (PUBMED_ID= 14762090), Milk fat yield (PUBMED_ID= 9691050), Production=Body weight (birth), (PUBMED_ID= 14677852), Chest depth (PUBMED_ID= 12605852), Body weight (birth), (PUBMED_ID= 20477797), Body weight (initial), (PUBMED_ID= 12805560),	ARL5A, CACNB4
HF	4	BovineHD0400009320	32656334	Exterior= Stature (PUBMED_ID= 16230715), Health= Immunoglobulin G level (PUBMED_ID= 21138580), Somatic cell score (PUBMED_ID= 9691050), Somatic cell score (PUBMED_ID= 14556700), Meat_and_Carcass= Tenderness score (PUBMED_ID= 11325189), Meat-to-bone ratio (PUBMED_ID= 18791160), Bone percentage (PUBMED_ID= 18791160), Carcass weight (PUBMED_ID= 11325189), Marbling score (PUBMED_ID= 19781039), Production= Average daily gain (PUBMED_ID= 11325189), Residual feed intake (PUBMED_ID= 18791150), Length of productive life (PUBMED_ID= 27889128),	RUNDC3B
HF	4	BovineHD0400015866	57768518	Exterior= Social separation--Vocalization (PUBMED_ID= 18784067), Social separation--Standing alert (PUBMED_ID= 18784067), Health= FMDV peptide-induced cell proliferation (PUBMED_ID= 21138580), Meat_and_Carcass= Marbling score (PUBMED_ID= 19781039), Longissimus muscle area (PUBMED_ID= 15537759), Marbling score (PUBMED_ID= 15537759), Milk= Milking speed (PUBMED_ID= 15377635), Milk protein yield (PUBMED_ID= 15377635), Milk fat percentage (PUBMED_ID= 9621249), Production= Body weight (weaning) (PUBMED_ID= 20477797), Body weight (slaughter) (PUBMED_ID= 22303340), Reproduction= Calving ease (PUBMED_ID= 18420641),	IMMP2L
HF	4	HAPLOTYPE	32641770-32656334	Exterior=Stature (PUBMED_ID= 16230715), Health=Immunoglobulin G level (PUBMED_ID= 21138580), Somatic cell score (PUBMED_ID= 14556700), Meat_and_Carcass=Tenderness score (PUBMED_ID= 11325189), Meat-to-bone ratio (PUBMED_ID= 18791160), Bone percentage (PUBMED_ID= 18791160), Carcass weight (PUBMED_ID= 11325189), Marbling score (PUBMED_ID= 19781039), Production=Average daily gain (PUBMED_ID= 11325189), Residual feed intake (PUBMED_ID= 18791150), Length of productive life (PUBMED_ID= 27889128),	RUNDC3B
HF	4	HAPLOTYPE	57701711-57701711	Exterior=Social separation--Vocalization (PUBMED_ID= 18784067), Social separation--Standing alert (PUBMED_ID= 18784067), Health=FMDV peptide-induced cell proliferation (PUBMED_ID= 21138580), Meat_and_Carcass=Marbling score (PUBMED_ID= 19781039), Longissimus muscle area (PUBMED_ID= 15537759), Marbling score (PUBMED_ID= 15537759), Milk=Milking speed (PUBMED_ID= 15377635), Milk protein yield (PUBMED_ID= 15377635), Milk fat percentage (PUBMED_ID= 9621249), Production=Body weight (weaning), (PUBMED_ID= 20477797), Body weight (slaughter), (PUBMED_ID= 22303340), Reproduction=Calving ease (PUBMED_ID= 18420641),	IMMP2L
HF	8	HAPLOTYPE	34351802-34400070	Exterior=Foot angle (PUBMED_ID= 21831322), Feet and leg conformation (PUBMED_ID= 21831322), Teat placement - front (PUBMED_ID= 21831322), Udder attachment (PUBMED_ID= 21831322), Rear leg placement - rear view (PUBMED_ID= 21831322), Teat placement - rear (PUBMED_ID= 21831322), Udder height (PUBMED_ID= 21831322), Stature (PUBMED_ID= 21831322), Strength (PUBMED_ID= 21831322), Udder cleft (PUBMED_ID= 21831322), Udder depth (PUBMED_ID= 21831322), Foot angle (PUBMED_ID= 21831322), Feet and leg conformation (PUBMED_ID= 21831322), Udder attachment (PUBMED_ID= 21831322), Stature (PUBMED_ID= 21831322), Strength (PUBMED_ID= 21831322), Udder depth (PUBMED_ID= 21831322), Health=Somatic cell score (PUBMED_ID= 18832229), Meat_and_Carcass=Fat thickness at the 12th rib (PUBMED_ID= 11325189), Marbling score	

				(PUBMED_ID= 20477797), Milk=Milking speed (PUBMED_ID= 29705414), Milk fat yield (PUBMED_ID= 21831322), Production=Body length (birth), (PUBMED_ID= 18791160), Body depth (PUBMED_ID= 21831322), PTA type (PUBMED_ID= 21831322), Net merit (PUBMED_ID= 21831322), Rump width (PUBMED_ID= 21831322), Body depth (PUBMED_ID= 21831322), PTA type (PUBMED_ID= 21831322), Rump width (PUBMED_ID= 21831322), Body weight (yearling), (PUBMED_ID= 30290764), Reproduction=Stillbirth (PUBMED_ID= 12613879), Stillbirth (maternal), (PUBMED_ID= 21831322),	
HF	15	HAPLOTYPE	82616506-82629156	Meat_and_Carcass=Carcass weight (PUBMED_ID= 20477797), Reproduction=Calving ease (PUBMED_ID= 20477797), Calf size (PUBMED_ID= 18420641), Age at puberty (PUBMED_ID= 22100599),	OR5A1, OR5AN1, OR5AN1M
HF	17	BovineHD1700007641	26596450	Health= PCVI minus PCVF (PUBMED_ID= 12805560), PCVF minus PCVM (PUBMED_ID= 12805560), PCV variance (PUBMED_ID= 12805560), Final packed red blood cell volume (PUBMED_ID= 12805560), Percentage decrease in PCV up to day 150 after challenge (PUBMED_ID= 12805560), Percentage decrease in PCV up to day 100 after challenge (PUBMED_ID= 12805560), Meat_and_Carcass= Marbling score (PUBMED_ID= 10764062), Milk= Milk alpha-casein percentage (PUBMED_ID= 19397519), Milk caproic acid content (PUBMED_ID= 29391528), Production= Average daily gain (PUBMED_ID= 17596127), Body weight (weaning) (PUBMED_ID= 20477797), Reproduction= Calving ease (PUBMED_ID= 20477797),	
HF	17	HAPLOTYPE	26596450-26603052	Health=PCVI minus PCVF (PUBMED_ID= 12805560), PCVF minus PCVM (PUBMED_ID= 12805560), PCV variance (PUBMED_ID= 12805560), Final packed red blood cell volume (PUBMED_ID= 12805560), Percentage decrease in PCV up to day 150 after challenge (PUBMED_ID= 12805560), Percentage decrease in PCV up to day 100 after challenge (PUBMED_ID= 12805560), Meat_and_Carcass=Marbling score (PUBMED_ID= 10764062), Milk=Milk alpha-casein percentage (PUBMED_ID= 19397519), Milk caproic acid content (PUBMED_ID= 29391528), Production=Average daily gain (PUBMED_ID= 17596127), Body weight (weaning), (PUBMED_ID= 20477797), Reproduction=Calving ease (PUBMED_ID= 20477797),	
HF	18	ARS-BFGL-NGS-107813	34260554	Exterior= Udder attachment (PUBMED_ID= 16230715), Udder height (PUBMED_ID= 16230715), Udder depth (PUBMED_ID= 16230715), Udder composite index (PUBMED_ID= 16230715), Health= Immunoglobulin G level (PUBMED_ID= 21138580), Meat_and_Carcass= Palmitic acid content (PUBMED_ID= 20477785), Omega-6 to omega-3 fatty acid ratio (PUBMED_ID= 20416790), Marbling score (PUBMED_ID= 27221246), Intramuscular fat (PUBMED_ID= 29163638), Milk= Milk yield (PUBMED_ID= 12487480), Production= Residual feed intake (PUBMED_ID= 18791150), Body weight (weaning) (PUBMED_ID= 20477797), Length of productive life (PUBMED_ID= 16734691), Reproduction= Dystocia (PUBMED_ID= 12613879), Stillbirth (PUBMED_ID= 19912419), Pregnancy rate (PUBMED_ID= 16734691),	BEAN1, CKLF, CMTM2, TK2
HF	21	BovineHD2100003533	13241858	Health= Clinical mastitis (PUBMED_ID= 14762087), Somatic cell count (PUBMED_ID= 16167984), Milk= Milk yield (PUBMED_ID= 12778594), Production= Body weight (birth) (PUBMED_ID= 15537758), Average daily gain (PUBMED_ID= 15537758), Reproduction= Stillbirth (PUBMED_ID= 19912419), Gestation length (PUBMED_ID= 19016677), Scrotal circumference (PUBMED_ID= 20477797),	
STAY	1	ARS-BFGL-BAC-14872	136386893	Exterior= Teat placement - front (PUBMED_ID= 16230715), Udder cleft (PUBMED_ID= 16230715), Meat_and_Carcass= Fat thickness at the 12th rib (PUBMED_ID= 20477797), Longissimus muscle area (PUBMED_ID= 20477797), Milk= Milk alpha-casein percentage (PUBMED_ID= 19397519), Milk fat yield (PUBMED_ID= 16533362), Milk fat yield (PUBMED_ID= 19841231), Production= Body weight (birth) (PUBMED_ID= 14677852), Body weight (yearling) (PUBMED_ID= 20477797), Body weight (weaning) (PUBMED_ID= 20477797), Veterinary treatments (PUBMED_ID= 19389971), Reproduction= Interval to first estrus after calving (PUBMED_ID= 19389971),	
STAY	21	BovineHD2100007401	25151974	Exterior= Udder width (PUBMED_ID= 16167984), Teat placement - front (PUBMED_ID= 16167984), Udder attachment (PUBMED_ID= 16167984), Teat length (PUBMED_ID= 16167984), Health= Clinical mastitis (PUBMED_ID= 14762087), Meat_and_Carcass= Fat thickness at the 12th rib (PUBMED_ID= 20477797), Carcass weight (PUBMED_ID= 20477797), Longissimus muscle area (PUBMED_ID= 20477797), Milk= Milk yield (PUBMED_ID= 12778594), Production= Body weight (weaning) (PUBMED_ID= 20477797), Body weight (birth) (PUBMED_ID= 15537758), Body weight (yearling) (PUBMED_ID= 20477797), PTA type (PUBMED_ID= 16167984), Reproduction= Stillbirth (PUBMED_ID= 19912419), Gestation length (PUBMED_ID= 19016677),	CTSH
STAY	28	BovineHD2800001953	6493973	Exterior= Udder cleft (PUBMED_ID= 12605852), Teat placement - front (PUBMED_ID= 12605852), Meat_and_Carcass= Fat thickness at the 12th rib (PUBMED_ID= 20477797), Longissimus muscle area (PUBMED_ID= 20477797), Carcass weight (PUBMED_ID= 20477797), Carcass weight (PUBMED_ID= 29103288), Milk= Milk lactose content (PUBMED_ID= 31178181), Milk mid-infrared spectra (PUBMED_ID= 31178181), Milk lactose content (PUBMED_ID= 31447150), Milk lactose content (PUBMED_ID= 29246110), Milk potassium content (PUBMED_ID= 33824377), Milk sodium content (PUBMED_ID= 33824377), Milk lactose content (PUBMED_ID= 31447150), Production= Body weight (initial) (PUBMED_ID= 12805560), Body weight (mean)	KCNK1

				(PUBMED_ID= 12805560), Body weight (weaning) (PUBMED_ID= 20477797), Body weight gain (PUBMED_ID= 19966163), Reproduction= Pregnancy rate (PUBMED_ID= 12605852), Gestation length (PUBMED_ID= 22034999),	
STAY	28	HAPLOTYPE	6480000-6493973	Exterior=Udder cleft (PUBMED_ID= 12605852), Teat placement - front (PUBMED_ID= 12605852), Meat_and_Carcass=Fat thickness at the 12th rib (PUBMED_ID= 20477797), Longissimus muscle area (PUBMED_ID= 20477797), Carcass weight (PUBMED_ID= 29103288), Milk=Milk protein yield (PUBMED_ID= 18650300), Milk lactose content (PUBMED_ID= 31178181), Milk mid-infrared spectra (PUBMED_ID= 31178181), Milk lactose content (PUBMED_ID= 29246110), Milk potassium content (PUBMED_ID= 33824377), Milk sodium content (PUBMED_ID= 33824377), Milk lactose content (PUBMED_ID= 31447150), Production=Body weight (initial), (PUBMED_ID= 12805560), Body weight (mean), (PUBMED_ID= 12805560), Body weight (weaning), (PUBMED_ID= 20477797), Body weight gain (PUBMED_ID= 19966163), Reproduction=Pregnancy rate (PUBMED_ID= 12605852), Gestation length (PUBMED_ID= 22034999),	KCNK1

[a] Chromosome; FS= Frame score; SC= Scrotal circumference; HF= Heifer fertility; STAY= Stayability.

Table S2. SNPs associated with reproductive traits and frame score with the models BAYESB, BAYESC and EMMAX in Simmental and Simbrah cattle.

Trait	Breed	Chr[a]	Position	SNP[b]	Model	PP[c]	P-value
FS[d]	Joint	4	59503146	BTB-00190917	BAYESB	0.08	
					BAYESC	0.07	
		5	118776285	BovineHD0500035146	BAYESB	0.32	
					BAYESC	0.30	
		6	37588140	BovineHD4100004588	BAYESC	0.04	
		7	467158	BovineHD0700000068	BAYESC	0.09	
		8	50833954	BovineHD0800015325	BAYESC	0.06	
			65744992	BovineHD0800019879	BAYESB	0.73	
					BAYESC	0.70	
			91458487	BovineHD0800027652	BAYESB	0.07	
					BAYESC	0.07	
			102704398	BovineHD4100007105	BAYESB	0.08	
					BAYESC	0.09	
		11	94731004	BovineHD1100027596	BAYESB	0.12	
					BAYESC	0.13	
		16	11855136	BovineHD1600003330	BAYESB	0.30	
					BAYESC	0.32	
		17	18626728	ARS-BFGL-NGS-118918	BAYESB	0.09	
					BAYESC	0.09	
			20844627	BovineHD1700006112	BAYESB	0.09	
					BAYESC	0.11	
			18626728	ARS-BFGL-NGS-118918	EMMAX		3.44E-06
		22	33624719	BovineHD2200009696	BAYESB	0.18	
					BAYESC	0.22	
			33760924	BovineHD2200009726	BAYESB	0.15	
					BAYESC	0.14	
			29120539	Hapmap53119-rs29018	BAYESB	0.10	
					BAYESC	0.10	
	Simbrah	3	104074474	BovineHD0300029985	BAYESB	0.12	
					BAYESC	0.14	
		17	24825598	BovineHD1700007095	BAYESB	0.12	
		18	47994061	ARS-BFGL-NGS-103183	BAYESB	0.10	
					BAYESC	0.16	
	Simmental	2	28349857	ARS-BFGL-NGS-60458	BAYESB	0.23	
					BAYESC	0.18	
			56674297	BTB-01145846	BAYESB	0.13	
					BAYESC	0.11	
		17	19461407	BTB-01087937	BAYESC	0.11	
		19	59345427	BovineHD1900017112	BAYESC	0.09	
			62590391	BovineHD1900018261	BAYESC	0.09	
		20	69702259	BovineHD2000020384	BAYESB	0.49	
					BAYESC	0.54	
					EMMAX		6.62E-06
SC[e]	Joint	1	24964189	BovineHD0100007238	BAYESB	0.22	
					BAYESC	0.24	
			69664149	BovineHD0100020122	BAYESC	0.06	
		3	14395023	BovineHD0300004685	BAYESB	0.08	
					BAYESC	0.08	

		6	32965679	BovineHD0600009601	BAYESB	0.46	
					BAYESC	0.46	
		10	18541772	ARS-BFGL-NGS-74837	BAYESB	0.22	
					BAYESC	0.22	
			22308257	BovineHD1000007178	BAYESB	0.13	
					BAYESC	0.14	
					EMMAX		8.50E-06
		17	15972230	BovineHD1700004678	BAYESB	0.17	
					BAYESC	0.18	
	Simbrah	6	32965679	BovineHD0600009601	BAYESB	0.16	
					BAYESC	0.20	
		10	18541772	ARS-BFGL-NGS-74837	BAYESB	0.10	
					BAYESC	0.10	
		17	7548169	BovineHD1700002151	BAYESC	0.08	
		23	23730493	BovineHD2300006211	BAYESC	0.12	
	Simmental	10	22308257	BovineHD1000007178	BAYESB	0.55	
					BAYESC	0.57	
					EMMAX		3.29E-06
		13	73602949	BovineHD1300021455	BAYESB	0.42	
					BAYESC	0.43	
FV[f]	Joint	4		ARS-BFGL-NGS-105821	BAYESC	0.07	
			57768518	BovineHD0400015866	BAYESC	0.10	
		17	26596450	BovineHD1700007641	BAYESB	0.32	
					BAYESC	0.34	
					EMMAX		3.19E-06
		18	34260554	ARS-BFGL-NGS-107813	BAYESB	0.16	
					BAYESC	0.18	
	Simbrah	17	26596450	BovineHD1700007641	BAYESB	0.15	
					BAYESC	0.15	
					EMMAX		6.46E-06
	Simmental	4	32656334	BovineHD0400009320	BAYESC	0.13	
		21	13241858	BovineHD2100003533	BAYESB	0.25	
					BAYESC	0.28	
					EMMAX		1.07E-05
STAY[g]	Joint	21	25151974	BovineHD2100007401	BAYESC	0.07	
	Simmental	1	136386893	ARS-BFGL-BAC-14872	BAYESB	0.13	
					BAYESC	0.15	
		28	6493973	BovineHD2800001953	BAYESB	0.26	
					BAYESC	0.32	
					EMMAX		4.87E-06

[a] Chromosome; [b] Single nucleotide polymorphism; [c] Posterior probabilities; [d] Frame score; [e] Scrotal circumference; [f] Heifer fertility; [g] Stayability.

Table S3. Haplotypes associated with reproductive traits and frame score with the models BAYESB, BAYESC and EMMAX in Simmental and Simbrah cattle.

Trait	Breed	Chr[a]	Position	SNPs[b]	Model	P-value	PP[c]
FS[d]	Joint	4	59489126-59503146	BovineHD0400016239, BTB-00190917	BAYESC		0.09
		8	65744992-65802549	BTB-01847877, BovineHD0800019879, BovineHD0800019885, BovineHD0800019890	BAYESB		0.76
					BAYESC		0.79
	Simbrah	3	104074474-104081372	BovineHD0300029985, BovineHD0300029986	BAYESB		0.22
					BAYESC		0.23
					EMMAX	1.37E-05	
	Simmental	1	60895445-60899823	BovineHD0100017414, ARS-BFGL-BAC-20015	BAYESB		0.43
					BAYESC		0.41
		2	28329020-28349857	Hapmap48633-BTA-118034, BovineHD0200008314, ARS-BFGL-NGS-60458	BAYESB		0.16
					BAYESC		0.14
		4	6351817-6418053	BovineHD0400001800, BovineHD0400001811, BovineHD0400001814, ARS-BFGL-NGS-66869	BAYESB		0.27
					BAYESC		0.34
		12	50678326-50689493	BTA-23832-no-rs, BovineHD1200014038	BAYESB		0.54
					BAYESC		0.66
SC[e]	Joint	19	43058602-43162697	BovineHD1900012356, BovineHD1900012359, BovineHD1900012364, BovineHD1900012372	BAYESC		0.08
		25	27577974-27654242	BovineHD2500007771, BovineHD2500007775, Hapmap44260-BTA-597, Hapmap41591-BTA-597	BAYESB		0.29
					BAYESC		0.34
					EMMAX	1.67E-05	
	Simbrah	2	5747611-5759652	BovineHD0200001651, BovineHD0200001656	BAYESB		0.48
					BAYESC		0.47
					EMMAX	1.12E-05	
		23	23730493-23745977	BovineHD2300006211, BovineHD2300006215	BAYESB		0.15
	Simmental	2	41538841-41567719	BovineHD0200040589, ARS-BFGL-BAC-2790, BovineHD0200012088, BovineHD0200012090	BAYESB		0.26
					BAYESC		0.23
		8	38363220-38366097	BovineHD0800011512, BovineHD0800011514	BAYESB		0.16
		9	15900318-15919300	BovineHD0900004308, BovineHD0900004311, BTB-01407863	BAYESB		0.16
					BAYESC		0.34
		13	73602949-73620495	BovineHD1300021455, BovineHD1300021459	BAYESB		0.20
FV[f]	Joint	2	44292504-44317544	BovineHD0200012875, BovineHD0200012878, BovineHD0200012883, BovineHD0200012884	BAYESB		0.07
					BAYESC		0.05
					EMMAX	4.73E-06	
		4	57701711-57701711	BovineHD0400015851, ARS-BFGL-NGS-105821	BAYESC		0.10
		15	82616506-82629156	BovineHD1500024589, BovineHD1500024595, BovineHD1500024597	BAYESC		0.09
		17	26596450-26603052	BovineHD1700007641, Hapmap43860-BTA-46675	BAYESB		0.58
					BAYESC		0.58
					EMMAX	1.27E-06	
	Simbrah	2	44282846-44313656	BovineHD0200012872, BovineHD0200012875, BovineHD0200012878, BovineHD0200012883, BovineHD0200012884, BovineHD0200012888	BAYESB		0.27
					BAYESC		0.06
					EMMAX	4.32E-06	

		8	34351802-34400070	BovineHD0800010236, Hapmap55107-rs29014632, BovineHD0800010244, BovineHD0800010247	BAYESB		0.15
					EMMAX	1.29E-05	
		28	9026425-9054402	BovineHD2800002746, ARS-BFGL-NGS-114215, BovineHD2800002756	EMMAX	1.38E-05	
	Simmental	4	32641770-32656334	BTB-01476087, BovineHD0400009320	BAYESB		0.49
					BAYESC		0.54
					EMMAX	1.18E-05	
STAY[g]	Simmental	28	6480000-6493973	BovineHD2800001945, BovineHD2800001953	BAYESB		0.28
					BAYESC		0.29
					EMMAX	5.65E-06	
		3	40032182-40154599	ARS-BFGL-NGS-114492, BTA-93165-no-rs, BovineHD0300012268, BTB-01436387, BovineHD0300012278	EMMAX	1.50E-05	

[a]Chromosome; [b]Single nucleotide polymorphism; [c]Posterior probabilities; [d]Frame score; [e]Scrotal circumference; [f]Heifer fertility; [g]Stayability.
